# Effect of Marine-Derived n-3 Polyunsaturated Fatty Acids on C-Reactive Protein, Interleukin 6 and Tumor Necrosis Factor α: A Meta-Analysis

**DOI:** 10.1371/journal.pone.0088103

**Published:** 2014-02-05

**Authors:** Kelei Li, Tao Huang, Jusheng Zheng, Kejian Wu, Duo Li

**Affiliations:** 1 Department of Food Science and Nutrition, Zhejiang University, Hangzhou, China; 2 APCNS Centre of Nutrition and Food Safety, Hangzhou, China; Max Delbrueck Center for Molecular Medicine, Germany

## Abstract

**Background:**

Previous studies did not draw a consistent conclusion about the effects of marine-derived n-3 polyunsaturated fatty acids (PUFAs) on fasting blood level of C-reactive protein (CRP), interleukin 6 (IL-6) and tumor necrosis factor α (TNF-α).

**Methods and Findings:**

A comprehensive search of Web of Science, PubMed, Embase and Medline (from 1950 to 2013) and bibliographies of relevant articles was undertaken. Sixty-eight RCTs with a total of 4601 subjects were included in the meta-analysis. Marine-derived n-3 PUFAs supplementation showed a lowering effect on Marine-derived n-3 PUFAs supplementation had a significant lowering effect on TNF-α, IL-6 and CRP in three groups of subjects (subjects with chronic non-autoimmune disease, subjects with chronic autoimmune disease and healthy subjects). A significant negative linear relationship between duration and effect size of marine-derived n-3 PUFAs supplementation on fasting blood levels of TNF-α and IL-6 in subjects with chronic non-autoimmune disease was observed, indicating that longer duration of supplementation could lead to a greater lowering effect. A similar linear relationship was also observed for IL-6 levels in healthy subjects. Restricted cubic spline analysis and subgroup analysis showed that the lowering effect of marine-derived n-3 PUFAs on CRP, IL-6 and TNF-α in subjects with chronic non-autoimmune disease became weakened when body mass index was greater than 30 kg/m^2^. The effect of marine-derived n-3 PUFAs from dietary intake was only assessed in subjects with chronic non-autoimmune disease, and a significant lowering effect was observed on IL-6, but not on CRP and TNF-α.

**Conclusions:**

Marine-derived n-3 PUFAs supplementation had a significant lowering effect on CRP, IL-6 and TNF-α level. The lowering effect was most effective in non-obese subjects and consecutive long-term supplementation was recommended.

## Introduction

Previous studies have shown that inflammation plays an important role in numerous chronic diseases, such as cardiovascular disease (CVD), diabetes, obesity-related diseases, and auto-immune diseases (e.g., rheumatoid arthritis) [1]–[4]. The process involves increased production of inflammatory factors, such as C-reactive protein (CRP), tumor necrosis factor α (TNF-α) and interleukin 6 (IL-6). CRP is an acute phase reactant protein that could increase 100 fold within 24 to 48 hours during an inflammatory process, and is synthesized and secreted mainly by hepatocytes [5]. Elevated CRP is an independent risk factor for CVD and diabetes [6], [7]. Previous studies have also shown a significant association between CRP and obesity [8], [9]. IL-6 and TNF-α are two cytokines involved in both acute and chronic inflammatory response, and one of their most important functions is the induction of acute phase reactant protein [10]. Strong evidence has also been provided for the association of IL-6 and TNF-α with obesity, CVD and diabetes [11]–[15].

Intake of n-3 polyunsaturated fatty acids (PUFAs) has been shown to have numerous beneficial effects on CVD, diabetes, and obesity-related diseases [16]–[18]. The protective effect of n-3 PUFAs against these diseases may be attributed to its anti-inflammatory function [19], [20]. However, some other studies found that n-3 PUFAs supplementation had no significant influence on the levels of inflammatory factors [21], [22]. The lack of consistency of results among different studies leads to a poor understanding of the association between n-3 PUFAs and inflammation. Two previous studies viewed the effect of marine-derived n-3 PUFAs on inflammatory makers, but no firm conclusion was drawn due to the contradiction of results from different studies [23], [24]. One previous meta-analysis assessed the effect of fish oil consumption on circulation levels of inflammatory markers, and found a significant lowering effect on CRP and IL-6 [25] in subjects with chronic heart failure. However, the subjects with other chronic diseases and healthy subjects were not included in this study, and the effect of marine-derived n-3 PUFAs from dietary intake was also not assessed. Therefore, we conducted a meta-analysis to systematically review the effect of marine-derived n-3 PUFAs from different sources (supplementation or dietary intake) on fasting blood levels of TNF-α, IL-6 and CRP in three groups of subjects (healthy subjects, subjects with chronic non-autoimmune disease or subjects with chronic autoimmune disease), and to obtain a pooled estimate of effect size.

## Methods

The protocol of the study was shown in Checklist S1.

### Data Sources and Study Selection

We searched Web of Science, Pubmed, Embase and Medline for the terms n-3 polyunsaturated fatty acid, omega-3 polyunsaturated fatty acid, ω-3 fatty acid, omega-3 fatty acid, polyunsaturated fatty acid, eicosapentaenoic acid (EPA), docosahexaenoic acid (DHA), or fish oil, combined with interleukin 6, interleukin, tumor necrosis factor, tumor necrosis factor alpha, TNF-α, cytokine, C-reactive protein, CRP, or inflammation. The search was restricted to studies published in any language from 1950 to 2013. For inclusion, studies had to fulfill the following criteria: had a randomized placebo controlled design; reported data on fasting blood levels of TNF-α, IL-6, or CRP; had a drop-out rate less than 30% [26]; recruited subjects with chronic disease (chronic autoimmune or non-autoimmune disease) or healthy subjects. Studies were excluded if subjects were diagnosed with acute disease; allocation of participants to the treatments was not randomized; data indispensable for a meta-analysis were not reported and still unavailable after contacting authors; studies reported a crossover design but not reported a wash-out period; the effect of n-3 PUFAs could not be separated from other active ingredients; studies did not have a placebo control group. Hand-searching of the bibliographic sections of all relevant articles and recent reviews was undertaken.

### Data Extraction and Quality Assessment

Study selection and data extraction were undertaken independently by two investigators, with discrepancies resolved by consensus. The data collected included the first author^’^s name, year of publication, sample size, age, BMI sex ratio (male/total subjects) and healthy status of subjects, intervention methods, study design (parallel-group, crossover or factorial), daily dose of n-3 PUFAs, duration of follow-up, data of fasting blood level of TNF-α, IL-6 and CRP, baseline levels of CRP, IL-6 and TNF-α, and mean changes of phospholipid n-3 PUFAs in plasma/serum.

Cochrane criteria was used to assess the quality of studies included in the present meta-analysis, including sequence generation, allocation concealment, blinding of participants, personnel and outcome assessors, incomplete outcome data and selective outcome reporting [27].

### Statistical Methods

Our study included studies in subjects with chronic disease and studies in healthy subjects. Considering the difference of pathogenesis between chronic autoimmune disease, which is an inappropriate immune response against substances and tissues normally present in the body, and other chronic disease (we called them chronic non-autoimmune diseases), two independent meta-analyses were conducted for the two types of chronic disease, respectively. Another independent meta-analysis was conducted for healthy subjects. We also separated studies using n-3 PUFAs from supplementation (seafood oils or highly purified n-3 PUFAs) as active treatment with those using n-3 PUFAs from dietary intake (fish intake), and included them into different meta-analyses.

As suggested in the Cochrane handbook for Systematic Reviews of Interventions [27], meta-analysis method is based on the assumption that data are normally distributed. However, many included studies showed that the data for CRP, IL-6 and TNF-α were skewed (expressed as data in log-transformed scale, geometric mean ± standard deviation (SD) or median (range or interquartile range)), and some studies expressed data as mean ± SD without reporting a test for distribution or even when data are skewed. Therefore, to eliminate potential bias induced by skewness of data, we transformed data of all studies into log (e)-transformed scale and then conducted data analysis [28]. If studies reported results as mean ± SD, the method reported by Higgins et al. [27], [29] was used to calculate the mean and SD of log-transformed data. If studies reported results as median and range, we firstly calculated the median and range for log-transformed data by taking the logarithms of median, upper and lower bounds of range for data on raw scale, and then calculated the mean and SD of log-transformed data using the method reported by Hozo et al. [30]. If studies reported result as median (interquartile range), we firstly calculated the median and interquartile range for log-transformed data by taking the logarithms of median, upper and lower bounds of interquartile range for data on raw scale; considering log-transformation can always substantially reduce skew [27], we used the median of log-transformed data to estimate its mean, and used interquratile range of log-transformed data divided by 1.35 to estimate its SD as suggested by Cochrane handbook for Systematic Reviews of Interventions [27].

For studies with a parallel-group or factorial design, mean changes and corresponding SDs from baseline to endpoint were used in data analysis. If SDs of changes were not reported, they were imputed based on SDs at baseline and endpoint by the method in a previous study [31]. For studies with a crossover design, mean difference between the levels of CRP, IL-6 or TNF-α at the end of two intervention periods was used in data analysis, as suggested by Cochrane handbook for Systematic Reviews of Interventions [27]. If a study involved two or more independent comparisons concerning the association between n-3 PUFAs and inflammatory markers (TNF-α, IL-6, or CRP), these comparisons were included in our meta-analysis as if they were from different studies [27]. For studies with two or more intervention groups sharing one control group, we separated the shared control group into two or more groups (the number was the same as intervention groups) and included these comparisons into meta-analysis as if they were from different studies [27]. If studies with a crossover design have two or more intervention periods (or placebo periods), only data of one intervention period (or placebo period) was included in analysis to eliminate unit-of-analysis error [27], but sensitivity analysis was conducted by replacing data from one intervention period (or placebo period) with another.

All data analyses were conducted in Stata/SE 11.0 software (StataCorp, College Station, TX). Weighted mean difference (WMD) was considered as the effect size. A random-effect model was used to pool study-specific effect sizes, and a P value <0.05 was considered to be statistically significant. Percentages of changes in geometric means of CRP, IL-6 and TNF-α from baseline to endpoint were also calculated based on WMDs using the following methods: estimating the ratio of geometric mean at endpoint to that at baseline based on WMD according to one study by Higgins et al. [29]; subtracting 1 from the ratio and multiplying the difference by 100%. Heterogeneity was assessed using the chi-square method. I^2^>50% indicated significant heterogeneity [32]. Subgroup analysis was undertaken to explore the sources of heterogeneity and their influence on effect size according to the difference in study design, placebo, duration, daily dose of n-3 PUFAs, and age, sex ratio (male/total subjects), baseline and body mass index (BMI) of subjects; the cut-off points for duration, daily dose,baseline and age were their medians; 0.5 was used as the cut-off point for sex ratio; 30 kg/m^2^ were used as the cut-off points for BMI. For meta-analysis in subjects with chronic diseases, subgroup analysis was also conducted according to difference in types of diseases. Meta-regression was conducted to explore whether subgroup difference was significant, and whether there was significant linear relationship (P for coefficient <0.05) between effect size and continuous confounding variables (duration, daily dose, age, sex ratio and BMI); the adjusted R^2^ indicated the percentage of between-study heterogeneity that could be explained by the confounding variables. If two or more confounding factors showed significant influence on the effect size (P for subgroup difference or coefficient <0.05), a joint test was conducted to explain heterogeneity caused by these confounding factors by including them into a single meta-regression model simultaneously [33]; the adjusted R^2^ of the overall model indicated the percentage of between-study heterogeneity that could be explained by these confounding factors [33]. If no significant linear relationship was observed between effect size and continuous confounding variables, restricted cubic spline analysis was used to test potential cubic relationship between them (3 knots) [34]. We also conducted sensitivity analysis to assessing the effect of bias on the pooled effect size by excluding studies having a high risk of bias for two or more validity criteria and then reanalyzing the remaining data. Publication bias was evaluated by funnel plot method. If significant publication bias was observed, trim-and-fill method was used to adjust the pooled effect size.

## Results

**Figure 1:**
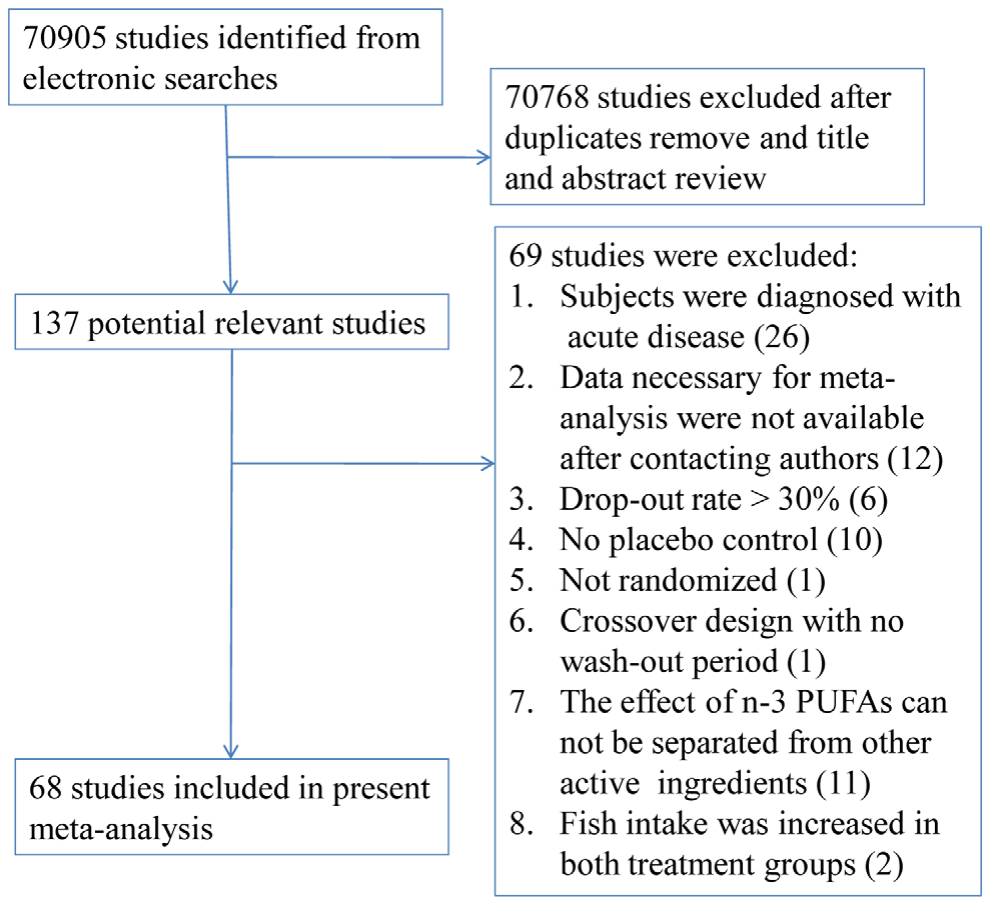
Flow chart for identifying eligible studies.

Figure 1 showed the process by which the included studies were identified. The electronic searches identified 70905 studies, of which 68 studies in 4601 subjects were included in our present study [19]–[22], [35]–[98].

### Characteristics of Included Studies

Characteristics of included studies are shown in Table 1 and Table S1. 48 studies including 61 independent comparisons involved patients with chronic non-autoimmune disease as subjects [19]–[21], [35]–[39], [42]–[44], [46]–[48], [50], [52], [53], [56], [58]–[63], [65], [67], [68], [70]–[78], [80], [83], [84], [86], [88], [89], [92], [94]–[98], 2 studies including 2 independent comparisons involved patients with chronic auto-immune disease as subjects [57], [93], and 17 studies including 22 independent comparisons involved healthy people as subjects [22], [40], [41], [49], [51], [54], [55], [64], [66], [69], [79], [81], [82], [85], [87], [90], [91]. In addition, one study including one independent comparison recruited both subjects with chronic non-autoimmune disease and subjects with chronic autoimmune disease [45]. Of the 48 studies in subjects with chronic non-autoimmune disease [19]–[21], [35]–[39], [42]–[44], [46]–[48], [50], [52], [53], [56], [58]–[63], [65], [67], [68], [70]–[78], [80], [83], [84], [86], [88], [89], [92], [94]–[98], 44 studies including 52 independent comparisons used n-3 PUFAs supplementation (seafood oil or highly purified n-3 PUFAs) as active treatment [19]–[21], [35]–[39], [42]–[44], [46]–[48], [50], [52], [53], [56], [58]–[63], [65], [67], [68], [70]–[78], [80], [83], [84], [86], [88], [89], [92], [97], and 5 studies including 11 independent comparisons used n-3 PUFAs from dietary intake (fish intake) as active treatment [94]–[98]. Studies in subjects with chronic auto-immune disease and in healthy subjects all used n-3 PUFAs supplementation as active treatment.

**Table 1:** Characteristics of included studies.

Study	T	C	Biomarkers	Duration	Design	EPA(g)	DHA (g)	Total n-3 (g)	No. of subjects	Age (yr)	Sex ratio (male/total)	BMI (kg/m^2^)	Disease
Barbosa et al.2003 [35]	Fish oil	Soy oil	CRP	2 mon	CO	2.7	1.8	4.5	9	40	0.22	UN	Ulcerative colitis
Bent et al.2011 [36]	Pudding fortified by fish oil	Pudding enriched in saff/ower oil	IL-6, TNF-α	12wk	P	0.7	0.46	1.3	25	70	0.89	UN	Autism spectrum disorder
Bowden et al.2009 [19]	Fish oil	Corn oil	CRP	6 mon	P	0.96	0.6	1.56	33	60.4	0.58	UN	End-stage renal disease
Bragt et al.2012 [37]	Fish oil	Cellulose and high-oleic sunflower oil	CRP, IL-6, TNF-α	6 wk	CO	1.7	1.2	3.7	10	52	0.5	33	Overweight and obesity
Browning et al.2007 A [38]	Fish oil	Oil rich in LA and oleic acid (2∶1)	CRP, IL-6	12 wk	CO	1.3	2.9	4.2	12	UN	0	32.8	Overweight
Browning et al.2007 B [38]	Fish oil	Oil rich in LA and oleic acid (2∶1)	CRP, IL-6	12 wk	CO	1.3	2.9	4.2	17	UN	0	28.7	Overweight
Chan et al.2002 [39]	Fish oil	Corn oil	CRP, IL-6, TNF-α	6 wk	P	1.8	1.56	3.36	24	53.5	1	33.6	Visceral obesity
Chiang et al.2012 [94]	Fatty fish diet	Control diet	CRP, IL-6, TNF-α	4 wk	CO	0.17	0.61	0.78	25	33	0.56	24.8	Normal to hyperlipidem-ia
De Mello et al.2009 A [95]	Fatty fish	Lean meat or skinless chicken	CRP, IL-6, TNF-α	8 wk	P	UN	UN	0.89	13	61.9	UN	26.9	Coronary heart disease
De Mello et al.2009 B [95]	Lean fish	Lean meat or skinless chicken	CRP, IL-6, TNF-α	8 wk	P	UN	UN	0.43	14	60.3	UN	27.5	Coronary heart disease
Derosa et al.2009 [44]	Fish oil	Sucrose, mannitol, and mineral salts	CRP	6 mon	P	1.64	1.36	3	326	51	0.49	26.1	Dyslipidemia
Derosa et al.2012 [43]	Fish oil	Sucrose, mannitol, and mineral salts	CRP, IL-6, TNF-α	6 mon	P	1.2	1.35	2.55	157	47	0.49	26.6	Dyslipidemia
Engler et al.2004 [46]	Algal oil	Soy/corn oil	CRP	6 wk	CO	NA	1.2	1.2	20	14	UN	21	Hyperlipide-mia
Faghihi et al.2012 [47]	Fish oil	Placebo	CRP	6 wk	P	UN	UN	0.6	41	33.92	0.76	24.7	Psychiatric disease
Faxen Irving et al. 2009 [48]	Fish oil	Corn oil	CRP, IL-6	6 mon	P	0.6	1.7	2.3	174	72.7	0.48	24.3	Alzheimer’s disease
Freund-Levi et al.2009 [21]	Fish oil	Corn oil	CRP, IL-6, TNF-α	6 mon	P	0.6	1.72	2.32	35	70.3	0.6	24.8	Alzheimer’s disease
Jones et al.2007 [52]	Fish oil	Sunflower oil (OA:LA = 1.1∶1) or olive oil	CRP, IL-6, TNF-α	4 wk	CO	1.7	3.7	5.4	21	54.19	0.46	25.93	Hypercholest-erolemia
Gammelmark et al. 2012 [50]	Fish oil	Olive oil	CRP, IL-6, TNF-α	6 wk	P	0.64	0.48	1.12	50	56.7	0.48	30.2	Overweight
Kabir et al.2007 [53]	Fish oil	Paraffin oil	IL-6, TNF-α	2 mon	P	1.08	0.72	1.8	26	55	0	30	Type 2 diabetes
Koh et al.2012 [56]	Fish oil	Placebo	CRP	2 mon	P	UN	UN	2	99	54.5	0.58	25.3	Hypertriglyc-eridemia
Kooshki et al.2011 [58]	Fish oil	Medium-chain triglyceride oils	CRP, IL-6, TNF-α	10 wk	P	1.24	0.84	2.08	34	50	0.62	19.8	Hemodialysis
Krebs et al.2006 [59]	Fish oil	Oil rich in LA and OA (2∶1)	CRP, IL-6, TNF-α	24 wk	P	1.3	2.9	4.2	67	44.7	0	35	Overweight
Krysiak et al.2011 a [60]	Fish oil	Placebo	CRP	12 wk	P	0.93	0.75	1.68	66	52.8	0.65	28.5	Hypertriglyc-eridemia
Krysiak et al.2011 b [61]	Fish oil	Placebo	CRP	90 d	P	0.93	0.75	1.68	54	53	0.63	28.5	Hypertriglyc-eridemia
Krysiak et al.2012 a [62]	Fish oil	Placebo	CRP	90 d	P	1.86	1.5	3.36	46	45	UN	UN	Hypertriglyc-eridemia
Krysiak et al.2012 b [63]	Fish oil	Placebo	CRP	12 wk	P	1.86	1.5	3.36	53	55	UN	UN	Hypertriglyc-eridemia
Lindqvist et al.2007 [96]	Herring	Pork/chicken	CRP	4 wk	CO	UN	UN	3.6	13	50.5	UN	32.6	Overweight and obesity
Mackay et al.2012 A [65]	Fish oil	Palm and soybean oils (80∶20)	CRP, IL-6	6 wk	CO	UN	UN	0.87	77	68.5	0.69	UN	Peripheral arterial disease
Mackay et al.2012 B [65]	Fish oil	Palm and soybean oils (80∶20)	CRP, IL-6	6 wk	CO	UN	UN	0.87	73	68.5	0.69	UN	Peripheral arterial disease
Madsen et al.2007 A [67]	Fish oil	Olive oil	CRP	8 wk	P	1.2	0.84	2.4	23	59	0.65	28	Chronic renal failure
Madsen et al.2007 B [67]	Fish oil	Olive oil	CRP	8 wk	P	1.2	0.84	2.4	23	59	0.65	28	Chronic renal failure
Malekshahi et al. 2012 [68]	Fish oil	High-linoleic sunflower oil	CRP, TNF-α	8 wk	P	1.56	0.83	2.73	84	54.2	0.5	27.6	Type 2 diabetes
Moertl et al.2011 A [71]	Fish oil	Gelatine	IL-6, TNF-α	3 mon	P	1.86	1.5	3.36	21	59.3	0.9	27.9	Chronic heart failure
Moertl et al.2011 B [71]	Fish oil	Gelatine	IL-6, TNF-α	3 mon	P	0.465	0.375	0.84	22	57.3	0.82	27.4	Chronic heart failure
Mohammadi et al. 2012 [72]	Fish oil	Liquid paraffin	CRP	8 wk	P	0.72	0.48	1.2	61	27.5	UN	28.8	Polycystic ovary syndrome
Mori et al.2003 A [74]	DHA	Olive oil	CRP, IL-6, TNF-α	6 wk	P	NA	4	4	25	61.2	0.74	30.3	Type 2 diabetes
Mori et al.2003 B [74]	EPA	Olive oil	CRP, IL-6, TNF-α	6 wk	P	4	NA	4	25	61.3	0.79	28.9	Type 2 diabetes
Mori et al.2009 [73]	Fish oil	Olive oil	CRP	8 wk	P	1.84	1.52	3.51	35	55.6	0.57	27.1	Chronic renal impairment
Munro et al.2012 [75]	Fish oil	Sunola oil	CRP, IL-6, TNF-α	14 wk	P	0.42	1.62	2.04	29	41.3	UN	32.8	Obesity
Murphy et al.2007 [76]	Food enriched in fish oil	Non-fortified food	CRP	6 mon	P	UN	UN	1	74	50.3	0.48	31.9	Overweight
Nodari et al.2009 [77]	Fish oil	Olive oil	IL-6, TNF-α	6 mon	P	0.54	0.9	1.44	44	63	0.91	UN	Idiopathic dilated cardiomyopa-thy
Nodari et al.2011 [78]	Fish oil	Olive oil	IL-6, TNF-α	12 mon	P	UN	UN	1.95	133	62.5	0.9	25.8	Chronic heart failure
Pooya et al.2010 [80]	Fish oil	High linoleic sunflower oil	CRP	2 mon	P	1.548	0.828	2.714	35	54.52	UN	26.89	Type 2 diabetes
Ramel et al.2010 AF [97]	Salmon	High oleic-sunflower oil	CRP, IL-6	8 wk	P	UN	UN	2.1	66	31.2	0	30.2	Overweight
Ramel et al.2010 AM [97]	Salmon	High oleic-sunflower oil	CRP, IL-6	8 wk	P	UN	UN	2.1	58	31.9	1	30.4	Overweight
Ramel et al.2010 BF [97]	Cod	High oleic-sunflower oil	CRP, IL-6	8 wk	P	UN	UN	0.3	69	30.9	0	30.2	Overweight
Ramel et al.2010 BM [97]	Cod	High oleic-sunflower oil	CRP, IL-6	8 wk	P	UN	UN	0.3	51	32.5	1	30.2	Overweight
Ramel et al.2010 CF [97]	Fish oil	High oleic-sunflower oil	CRP, IL-6	8 wk	P	UN	UN	1.3	99	31.3	0	30	Overweight
Ramel et al.2010 CM [97]	Fish oil	High oleic-sunflower oil	CRP, IL-6	8 wk	P	UN	UN	1.3	61	31.8	1	29.8	Overweight
Sabour et al.2012 [83]	Fish oil	Placebo	IL-6, TNF-α	4 mon	P	0.07	0.44	0.51	75	39.3	0.83	19.7	Spinal cord injury
Saifullah et al.2007 [84]	Fish oil	Soybean/corn oil	CRP	8 wk	P	0.68	0.34	1.02	23	57.7	0.78	UN	Hemodialysis
Skulas-Ray et al. 2011 [86]	Fish oil	Corn oil	CRP, IL-6, TNF-α	8 wk	CO	2.04	1.36	3.4	26	44.3	0.88	29	Moderate hypertriglyce-ridemia
Thusgaard et al. 2009 [88]	Fish oil	Corn oil	CRP	12 wk	P	1.84	1.52	3.36	48	45	0.78	24.7	HIV infected
Tierney et al.2011 [89]	Fish oil	High oleic-sunflower oil	CRP, IL-6, TNF-α	12 wk	P	UN	UN	1.24	206	54.7	UN	32.5	Metabolic syndrome
Wong et al.2010 [92]	Fish oil	Olive oil	CRP	12 wk	P	1.68	1	2.68	97	60.1	0.44	25.8	Type 2 diabetes
Daud et al.2012 [42]	Fish oil	Olive oil	CRP	6 mon	P	1.8	0.6	2.4	55	58.5	0.51	27.6	Hemodialysis
Zhang et al.2012 A [98]	Salmon	Pork/beef/chicken/fish	CRP, IL-6, TNF-α	8 wk	P	0.61	1.07	1.69	43	55.3	0	26.3	Dyslipidaem-ia
Zhang et al.2012 B [98]	Herring	Pork/beef/chicken/fish	CRP, IL-6, TNF-α	8 wk	P	0.7	0.97	1.67	39	55.8	0	27.3	Dyslipidaem-ia
Zhang et al.2012 C [98]	Pompano	Pork/beef/chicken/fish	CRP, IL-6, TNF-α	8 wk	P	0.14	0.94	1.07	44	56.5	0	26.5	Dyslipidaem-ia
Zhao et al.2009 [20]	Fish oil	Placebo	CRP, IL-6, TNF-α	3 mon	P	0.36	0.24	0.6	75	72.5	0.73	24.4	Chronic heart failure
Mocking et al.2012 [70]	EPA	Rapeseed oil and medium chain triglyceride	CRP, IL-6, TNF-α	12 wk	P	0.9	0	0.9	24	54	0.48	UN	Diabetes mellitus and co-morbid depression
Deutsch et al.2007 [45]	Krill oil	Microcrystalli-ne cellulose	CRP	30 d	P	0.05	0.03	0.08	87	54.95	0.34	UN	CVD or arthritis
Kolahi et al.2010 [57]	Fish oil	Placebo	CRP, TNF-α	3 mon	P	0.18	0.12	0.3	83	50	0	25.4	Rheumatoid arthritis
Wright et al.2008 [93]	Fish oil	Olive oil	CRP	24 wk	P	1.8	1.2	3	60	48.1	0.07	25.6	Systemic lupus erythematosus
Fujioka et al.2006 [49]	Drinks enriched in fish oil	Drinks enriched in olive oil	CRP	12 wk	P	0.6	0.26	0.86	141	37.9	0.42	22.1	Healthy
Lenn et al.2002 [64]	Fish oil	Western fat blend and or wheat flour	IL-6, TNF-α	30 d	P	UN	UN	1.8	10	21.9	0.77	24	Healthy
Madsen et al.2003 A [66]	Fish oil	Olive oil	CRP	12 wk	P	3	2.9	6.6	30	38.3	0.57	24.4	Healthy
Madsen et al.2003 B [66]	Fish oil	Olive oil	CRP	12 wk	P	0.9	0.8	2	30	37.7	0.6	24.7	Healthy
Mann et al.2010 A [69]	Fish oil	Sunola oil	CRP	14 d	P	0.21	0.81	1.05	14	29.7	0.44	UN	Healthy
Mann et al.2010 B [69]	Seal oil	Sunola oil	CRP	14 d	P	0.34	0.45	1.02	13	30.5	0.22	UN	Healthy
Ottestad et al.2012 [79]	Fish oil	High-oleic sunflower oil	CRP	7 wk	P	0.72	0.89	1.76	34	25	0.28	22.8	Healthy
Pot et al.2009 [81]	Fish oil	High-oleic sunflower oil	IL-6, TNF-α	12 wk	P	0.7	0.56	1.52	77	58.7	0.51	26.5	Healthy
Rizza et al.2009 [82]	Fish oil	Olive oil	CRP, IL-6, TNF-α	12 wk	P	1.09	0.91	2	50	31.1	0.5	26.2	Healthy
Sanders et al.2006 [22]	Algal oil	Olive oil	CRP	4 wk	P	0	1.5	2.1	79	32.5	0.51	23.8	Healthy
Shahbakhti et al. 2004 [85]	EPA	Oleic acid	IL-6, TNF-α	3 mon	P	4	0	4	14	47.5	0	UN	Healthy
Theobald et al.2007 [87]	Algal oil	Olive oil	CRP, IL-6	3 mon	CO	0	0.7	0.7	38	48.7	0.5	24	Healthy
Vega-Lopez et al. 2004 [90]	Fish oil	Placebo	CRP	12 wk	P	0.6	0.9	1.5	40	31.5	UN	26.1	Healthy
Watanabe et al.2009 [91]	Fish oil	Olive oil	CRP	4 wk	CO	1.26	0.54	1.8	17	50.1	1	24.3	Healthy
Geelen et al.2004 [51]	Fish oil	High-oleic sunflower oil	CRP	12 wk	P	0.7	0.56	1.52	83	60	0.51	UN	Healthy
Kiecolt-Glaser et al. 2012 A [55]	Fish oil	Mixture of palm, olive, soy, canola, and coco butter oils	IL-6, TNF-α	4 mon	P	2.09	0.35	2.5	69	51	0.32	UN	Healthy
Kiecolt-Glaser et al. 2012 B [55]	Fish oil	Mixture of palm, olive, soy, canola, and coco butter oils	IL-6, TNF-α	4 mon	P	1.04	0.17	1.25	69	51.1	0.33	UN	Healthy
Ciubotaru et al.2003 A [40]	Fish oil	Safflower oil	CRP, IL-6	5 wk	P	1.18	1	2.33	11	60	0	25	Healthy
Ciubotaru et al.2003 B [40]	Fish oil	Safflower oil	CRP, IL-6	5 wk	P	0.59	0.5	1.22	15	60	0	17.1	Healthy
Kiecolt-Glaser et al. 2011 [54]	Fish oil	Mixture of palm, olive, soy, canola, and coco butter oils	IL-6, TNF-α	12 wk	P	2.09	0.35	2.5	68	23.7	0.56	UN	Healthy
Damsgaard et al. 2008 A [41]	Fish oil	Olive oil	CRP, IL-6	8 wk	F	1.8	1.1	3.1	33	25.6	1	22.5	Healthy
Damsgaard et al. 2008 B 28 [41]	Fish oil	Olive oil	CRP, IL-6	8 wk	F	1.8	1.1	3.1	29	25.2	1	23.1	Healthy

T, intervention in treatment group; C, intervention in control group; P, parallel group design; CO, crossover design; F, factorial design; UN, unclear.

Studies marked with different capital letters were independent comparisons from the same study; studies marked with different minuscules were different studies with the same first author and published in the same year.

### Quality Assessment

Incomplete outcome data (attrition bias) and blinding of participants and personnel (performance bias) were the major potential sources of bias (Figure S1). In many trails, it was unclear whether randomized sequence could be foreseen by participants and trialists (allocation concealment) and whether assessors were blinded to intervention assignment (detection bias) (Figure S1). Twenty-six studies have a risk of bias in one category [19], [20], [37], [38], [43], [44], [48], [52], [53], [56], [60], [61], [70], [73], [75], [79], [80], [83], [84], [86], [88], [89], [94], [96]–[98], and one study [95] has a risk of bias in two categories (Figure S2).

### Effects of Marine-Derived N-3 PUFAs Supplementation on CRP, IL-6 and TNF-α in Subjects with Chronic Non-Autoimmune Disease

**Figure 2:**
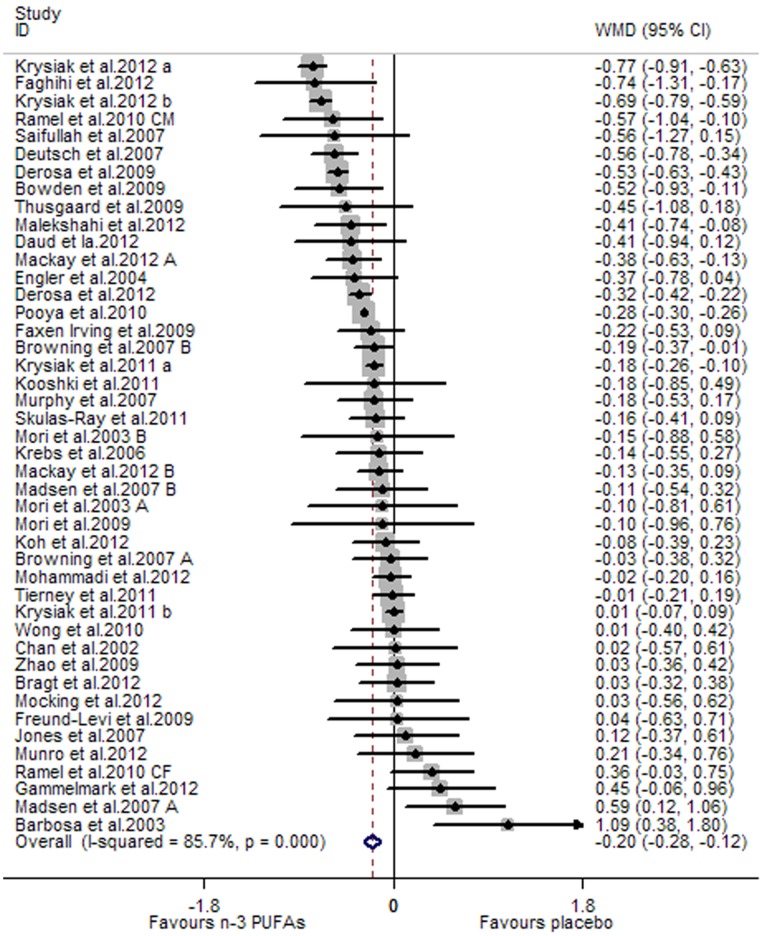
Pooled effect size of n-3 PUFAs supplementation on CRP in chronic non-autoimmune disease. **WMD, weighted mean difference.**

**Figure 3:**
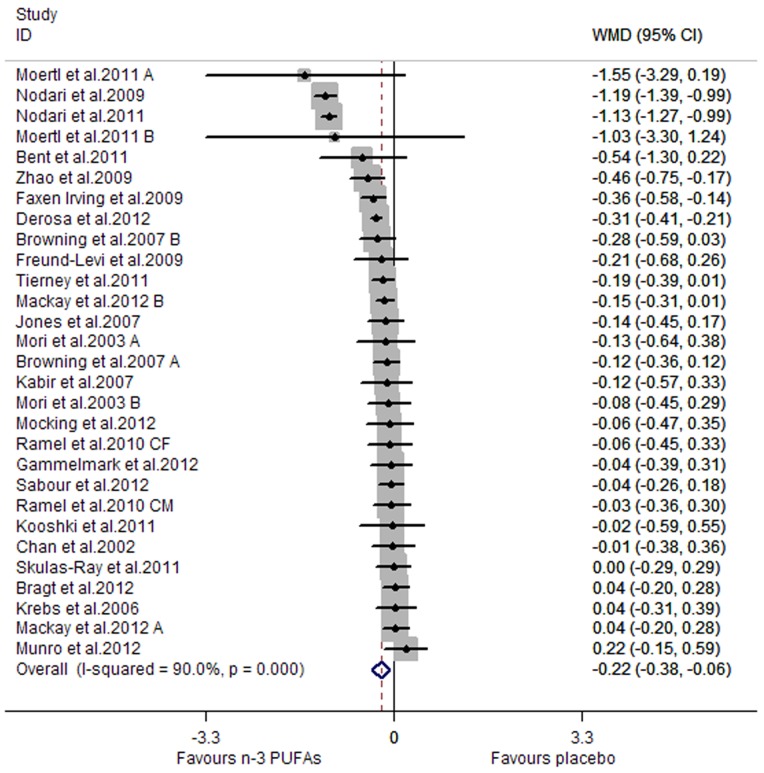
Pooled effect size of n-3 PUFAs supplementation on IL-6 in chronic non-autoimmune disease. **WMD, weighted mean difference.**

**Figure 4:**
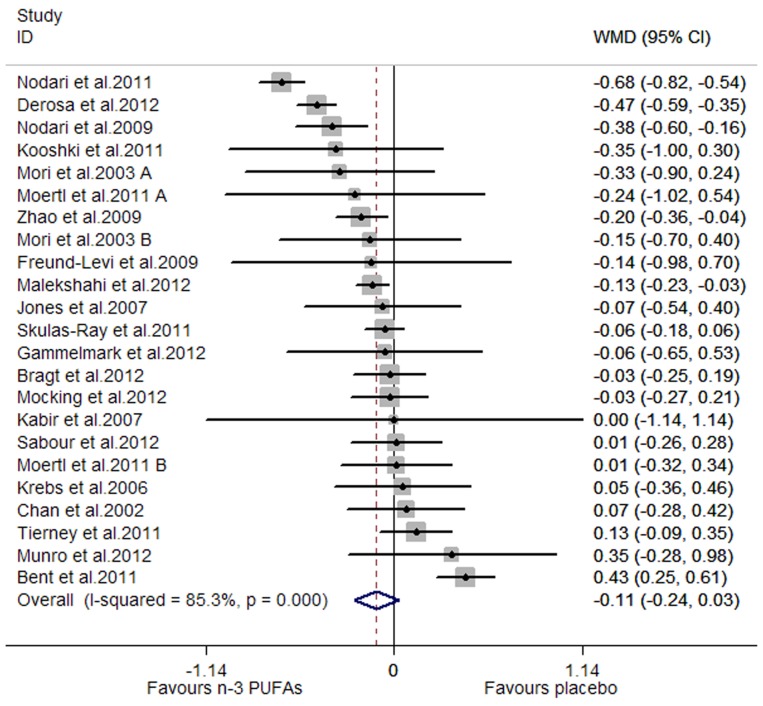
Pooled effect size of n-3 PUFAs supplementation on TNF-α in chronic non-autoimmune disease. **WMD, weighted mean difference.**

A significant lowering effect of marine-derived n-3 PUFA supplementation on CRP and IL-6 was observed: the pooled effect sizes were −0.20 (95% CI, −0.28 to −0.12; P = 0.000) (Figure 2) and −0.22 (95% CI, −0.38 to −0.06; P = 0.008) (Figure 3), respectively. The overall percentages of changes (in geometric mean) were −18.13% (95% CI, −24.42% to −11.31%) and −19.75% (95% CI, −31.61% to −5.82%), respectively. The pooled effect size for TNF-α was not significant (Figure 4). Significant heterogeneity was observed among the three groups of studies: the I^2^ values were 85.7%, 90.0% and 85.3%, respectively. One study assessing the effect on CRP recruited both subjects with chronic non-autoimmune disease and subjects with chronic autoimmune disease [45], and after excluding this study the result concerning CRP did not change significantly. One study assessing the effect on CRP, IL-6 and TNF-α used two kinds of placebo (sunflower oil and olive oil) [52], and when we replaced data from one kind of placebo with data from the other the result concerning the three inflammatory markers did not change significantly.

Results of subgroup analysis were shown in Table 2. Fatty acid composition of placebo and BMI of subjects had a significant influence on the effect size on CRP: significant lowering effect on CRP was observed only when oils mainly comprised of linoleic acid (LA) were used as placebo or subjects had a BMI less than 30 kg/m^2^ (P for subgroup difference were 0.033 and 0.014, respectively); the overall percentages of changes (in geometric mean) in the two subgroups were −19.75% (95% CI, −28.11% to −10.42%) and −18.13% (95% CI −24.42% to −10.42%), respectively; joint test for the two covariates (as categorical variables) showed that their variation could help explain 45.37% heterogeneity. Duration, age and baseline level of IL-6 had a significant influence on the effect size on IL-6: significant lowering effect was observed only when studies had a longer duration (≥ median), or recruited older subjects (age ≥ median) (P for subgroup difference were 0.022 and 0.014, respectively), and the overall percentages of changes (in geometric mean) in the two subgroups were −30.23% (95% CI, −45.66% to −11.31%) and −33.63% (95% CI, −50.84% to −10.42%), respectively; the lowering effect on IL-6 was significantly greater in subjects with a higher baseline level (≥ median) than those with a lower one (< median) (P for subgroup difference was 0.049), and the overall percentages of changes (in geometric mean) in the two subgroups were −32.29% (95% CI, −50.34% to −6.76%) and −12.19% (95% CI, −20.55% to −1.98%), respectively; joint test for duration, age and baseline of IL-6 (as categorical variables) showed that their variation could help explain 46.56% heterogeneity. BMI of subjects had a significant influence on the effect size on TNF-α: significant lowering effect of marine-derived n-3 PUFAs on TNF-α was only observed in subjects with a BMI <30 kg/m^2^ (P value for subgroup difference was 0.049), and its variation (as categorical variable) could help explain 23.54% heterogeneity; the overall percentage of changes (in geometric mean) in this subgroup was −19.75% (95% CI, −31.61% to −5.82%).

**Table 2:** The effect of marine-derived n-3 PUFAs supplementation on CRP, IL-6 and TNF-α in subjects with chronic no-autoimmune disease (data were log-transformed before analysis).

	CRP				IL-6				TNF-α			
Subgroup analysis	N	WMD (95% CI)	I^2^ (%)	P	N	WMD (95% CI)	I^2^ (%)	P	N	WMD (95% CI)	I^2^ (%)	P
Overall	44	−0.20 (−0.28, −0.12)	85.7	0.000	29	−0.22 (−0.38, −0.06)	90.0	0.008	23	−0.11 (−0.24, 0.03)	85.3	0.121
Study design												
Parallel	35	−0.22 (−0.32, −0.13)	87.6	0.000	22	−0.27 (−0.47, −0.06)	91.0	0.011	20	−0.11 (−0.27, 0.04)	86.7	0.156
Crossover	9	−0.11 (−0.26, 0.05)	57.8	0.190	7	−0.09 (−0.18, 0.00)	0.0	0.053	3	−0.05 (−0.15, 0.05)	0.0	0.295
Major fatty acid in placebo												
Linoleic acid	14	−**0.22 (−0.33, −0.11)**	44.9	0.000	8	−0.17 (−0.29, −0.05)	11.5	0.005	6	0.06 (−0.15, 0.26)	83.7	0.599
Oleic acid	13	**0.03 (−0.13, 0.20)**	43.2	0.704	10	−0.27 (−0.65, 0.11)	95.4	0.011	8	−0.17 (−0.46, 0.12)	87.5	0.243
Others	10	−**0.27 (−0.41, −0.12)**	77.9	0.000	9	−0.16 (−0.29, −0.04)	38.0	0.257	7	−0.19 (−0.42, 0.05)	65.2	0.122
Not reported	7	−0.34 (−0.62, −0.06)	96.7	0.017	2	−0.24 (−0.65, 0.17)	80.4	0.003	2	−0.13 (−0.32, 0.07)	41.0	0.207
Healthy status of subjects#												
Presence or high risk of CVD	31	−0.22 (−0.30, −0.13)	88.4	0.000	24	−0.22 (−0.40, −0.03)	91.5	0.021	19	−0.15 (−0.28, −0.02)	81.8	0.024
Chronic renal disease	7	−0.17 (−0.50, 0.15)	59.6	0.300	1	−0.02 (−0.59, 0.55)	NA	0.945	1	−0.35 (−1.00, 0.30)	NA	0.289
Psychiatric disease	4	−0.24 (−0.54, 0.07)	33.0	0.134	4	−0.30 (−0.47, −0.12)	0.0	0.001	3	0.15 (−0.24, 0.55)	80.5	0.444
Other chronic diseases	3	0.17 (−0.51, 0.85)	82.1	0.631	1	−0.04 (−0.26, 0.18)	NA	0.716	1	0.01 (−0.26, 0.28)	NA	0.943
Duration (week)												
< median	17	−0.16 (−0.27, −0.05)	66.9	0.004	13	−**0.06 (**−**0.14, 0.02)**	0.0	0.154	10	−0.09 (−0.16, −0.03)	0.0	0.006
≥ median	18	−0.21 (−0.35, −0.07)	91.1	0.004	16	−**0.36 (**−**0.61,** −**0.12)**	93.0	0.003	13	−0.10 (−0.32, 0.12)	91.2	0.356
Daily dose of total n-3 PUFAs (g)												
< median	23	−0.16 (−0.25, −0.06)	65.4	0.001	14	−0.32 (−0.61, −0.03)	94.1	0.033	11	−0.06 (−0.31, 0.20)	91.4	0.657
≥ median	21	−0.23 (−0.36, −0.11)	88.6	0.000	15	−0.13 (−0.24, −0.03)	42.6	0.012	12	−0.15 (−0.29, −0.01)	67.8	0.032
Daily dose of EPA (g)												
< median	18	−0.13 (−0.25, −0.01)	74.9	0.029	11	−0.30 (−0.61, 0.02)	89.2	0.066	10	−0.03 (−0.24, 0.18)	79.3	0.769
≥ median	18	−0.25 (−0.39, −0.12)	89.5	0.000	12	−0.13 (−0.24, −0.02)	36.8	0.022	11	−0.14 (−0.28, 0.00)	70.5	0.058
Not reported	8	−0.17 (−0.36, 0.01)	63.2	0.059	6	−0.26 (−0.71, 0.19)	96.5	0.259	2	−0.28 (−1.07, 0.51)	97.4	0.490
Daily dose of DHA (g)												
< median	18	−0.16 (−0.27, −0.05)	82.4	0.004	11	−0.27 (−0.60, 0.07)	89.9	0.116	10	−0.02 (−0.19, 0.15)	74.8	0.816
≥ median	18	−0.21 (−0.37, −0.06)	85.7	0.006	12	−0.16 (−0.28, −0.05)	42.4	0.005	11	−0.14 (−0.31, 0.02)	73.4	0.094
Not reported	8	−0.17 (−0.36, −0.01)	63.2	0.059	6	−0.26 (−0.71, 0.19)	96.5	0.259	2	−0.28 (−1.07, 0.51)	97.4	0.490
Mean age (year)												
< median	21	−0.17 (−0.31, −0.03)	88.8	0.016	13	**−0.08 (−0.18, 0.02)**	43.8	0.123	11	−0.09 (−0.23, 0.04)	74.7	0.174
≥ median	21	−0.21 (−0.34, −0.08)	82.5	0.001	14	**−0.41 (−0.71, −0.11)**	93.0	0.008	12	−0.14 (−0.40, 0.13)	90.0	0.315
Not reported	2	−0.16 (−0.32, −0.00)	0.0	0.050	2	**−**0.18 (−0.37, 0.11)	0.0	0.064	0	NA	NA	NA
Sex ratio (male/total subjects)												
<0.5	14	−0.11 (−0.27, 0.06)	81.8	0.199	10	−0.22 (−0.31, −0.14)	10.6	0.000	5	−0.15 (−0.31, 0.01)	68.7	0.069
≥0.5	23	−0.17 (−0.27, −0.07)	52.0	0.001	17	−0.30 (−0.58, −0.02)	93.4	0.036	13	−0.13 (−0.36, 0.10)	90.9	0.261
Not reported	7	−0.31 (−0.54, −0.09)	95.4	0.006	2	−0.02 (−0.41, 0.38)	72.6	0.934	2	0.15 (−0.05, 0.36)	0.0	0.141
Mean BMI												
<30 kg/m^2^	25	**−0.20 (−0.28, −0.11)**	80.0	0.000	14	−0.30 (−0.54, −0.06)	91.0	0.016	12	**−0.22 (−0.38, −0.06)**	85.1	0.007
≥30 kg/m^2^	10	**0.03 (−0.09, 0.14)**	0.0	0.634	10	−0.06 (−0.16, 0.04)	0.0	0.230	8	**0.04 (−0.08, 0.16)**	0.0	0.515
Not reported	9	−0.38 (−0.60, −0.17)	47.1	0.001	5	−0.38 (−0.92, 0.17)	85.5	0.175	3	0.01 (−0.48, 0.50)	94.0	0.969
Baseline (log-transformed)												
< median	21	−0.15 (−0.31, 0.01)	86.9	0.059	13	**−0.13 (−0.23, −0.02)**	47.4	0.015	10	−0.01 (−0.09, 0.08)	0.0	0.894
≥ median	21	−0.23 (−0.33, −0.13)	84.0	0.000	14	**−0.39 (−0.70, −0.07)**	92.8	0.015	11	−0.22 (−0.43, −0.00)	91.6	0.047
Not reported	2	0.06 (−0.23, 0.35)	0.0	0.678	2	−0.02 (−0.21, 0.16)	0.0	0.796	2	−0.04 (−0.23, 0.16)	0.0	0.712

N, number of included studies (or comparisons); NA, not associated with this item; CVD, cardiovascular disease.

<?ENTCHAR num?> Subjects with high risk of CVD included overweight (or obese) subjects, subjects with dislipidemia, subjects with diabetes, and subjects with metabolic syndrome; subjects in the study by Mocking et al. in 2012 were diagnosed with both diabetes mellitus and depression simulaneously, so this study was included in both subgroup “presence or high risk of CVD” and subgroup “psychiatric disease” without calculating the overall effect size for all the studies.

Unit for CRP: mg/L; unit for IL-6 and TNF-α: pg/mL.

Data expressed in bold indicated that subgroup difference was significant (P for subgroup difference <0.05), and subgroup “not reported” was not included in the significance test for subgroup difference.

**Figure 5:**
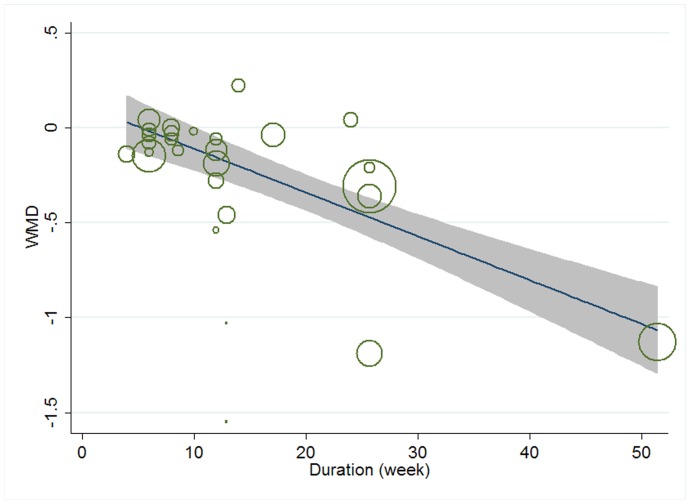
Meta-regression for duration and effect size (n-3 PUFAs supplementation on IL-6 in chronic non-autoimmune disease). WMD, weighted mean difference.

**Figure 6:**
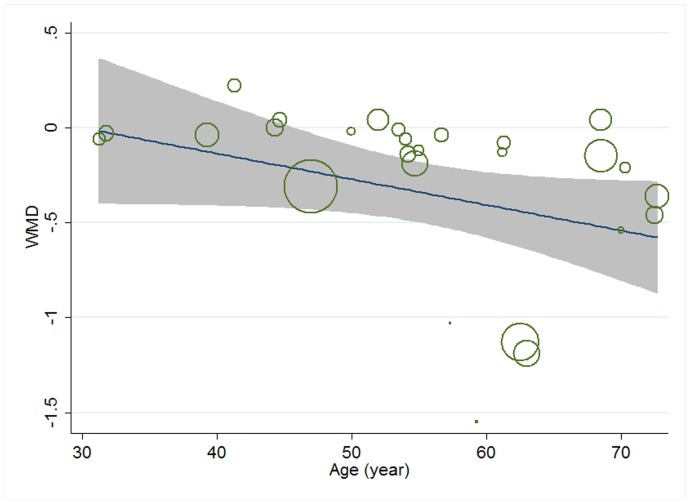
Meta-regression for age and effect size (n-3 PUFAs supplementation on IL-6 in chronic non-autoimmune disease). WMD, weighted mean difference.

**Figure 7:**
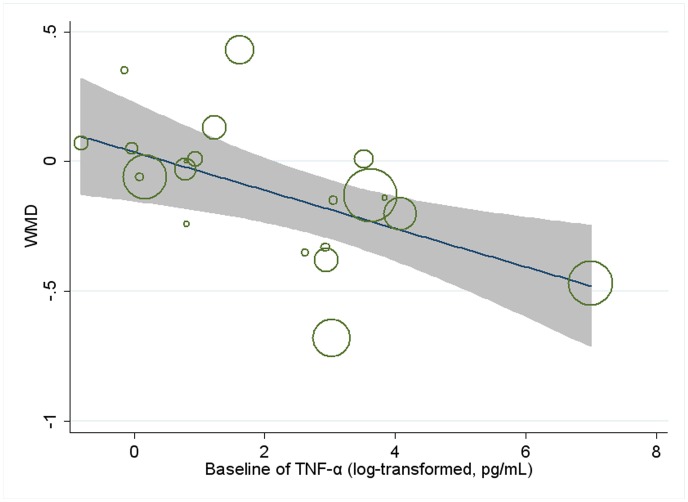
Meta-regression for baseline of TNF-α and effect size (n-3 PUFAs supplementation on TNF-α in chronic non-autoimmune disease). WMD, weighted mean difference.

**Figure 8:**
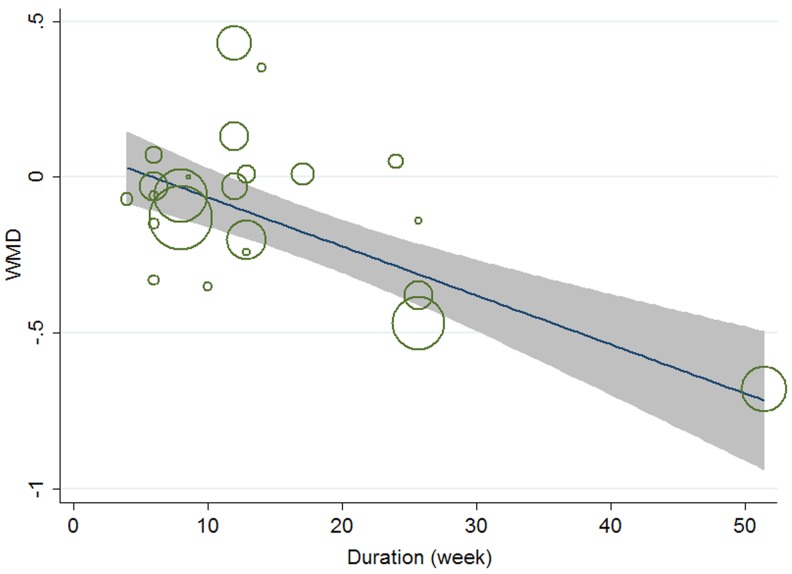
Meta-regression for duration and effect size (n-3 PUFAs supplementation on TNF-α in chronic non-autoimmune disease). WMD, weighted mean difference.

Meta-regression was conducted to assess the potential linear relationship between effect size and continuous covariates (baseline, duration, age, sex ratio (male/total subjects), BMI, and daily dose of EPA, DHA and total n-3 PUFAs). No significant result was observed for CRP. A significant linear relationship was observed between effect size on IL-6 and duration as well as age, and the coefficients were −0.023 (95% CI, −0.032 to −0.014; P = 0.000) (Figure 5) and −0.013 (95% CI, −0.025 to −0.000; P = 0.042) (Figure 6), respectively, indicating that the lowering effect was more effective when a longer duration was used or older subjects were recruited; the percentages of changes (in geometric mean) with 1 unit increase of duration (week) and age (year) were −2.27% (95% CI, −3.15% to −1.39%) and −1.29% (−2.47% to −0.0%); joint test for duration and age (as continuous variables) showed that their variation could help explain 67.08% heterogeneity; no significant linear relationship was observed between effect size on IL-6 and other continuous confounding variables. A significant linear relationship was observed between effect size on TNF-α and baseline level of TNF-α as well as duration, and the coefficients were −0.081 (95% CI, −0.145 to −0.017; P = 0.016) (Figure 7) and −0.015 (95% CI, −0.024 to −0.007; P = 0.001) (Figure 8), indicating that a higher baseline level of TNF-α or a longer duration could lead to a greater lowering effect; the percentages of changes (in geometric mean) with 1 unit increase of baseline level (log-transformed, pg/mL) and duration (week) were −7.78% (95% CI, −13.50% to −1.69%) and −1.49% (−2.37% to −0.70%); joint test for baseline of TNF-α and duration (as continuous variable) could help explain 63.21% heterogeneity; the significant linear relationship between duration and effect size demonstrated that marine-derived n-3 PUFAs supplementation had a significant lowering effect on TNF-α in subjects with chronic non-autoimmune disease although the pooled effect size was not significant; no significant linear relationship was observed between effect size on TNF-α and other continuous confounding variables.

**Figure 9:**
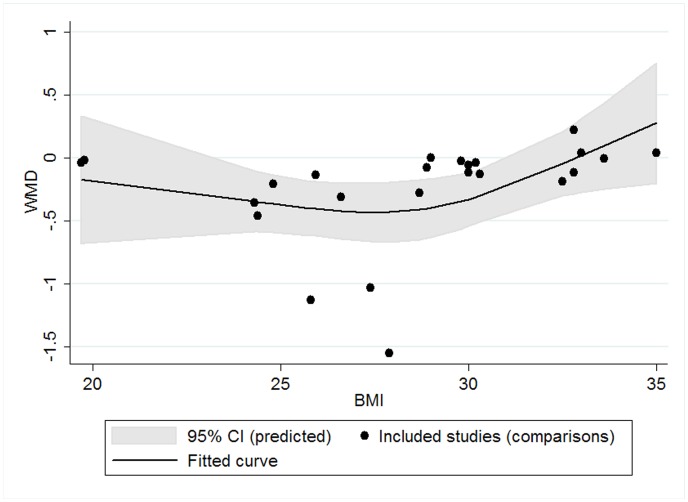
Cubic relationship between BMI and effect size (n-3 PUFAs supplementation on IL-6 in chronic non-autoimmune disease). WMD, weighted mean difference.

Restricted cubic spline analysis was used to assess potential cubic relationship between effect size and continuous covariates (as mentioned above). No significant result was observed for CRP and TNF-α. We found a significant cubic relationship between BMI of subjects and effect size on IL-6 (P for linearity = 0.047): the lowering effect did not change significantly with BMI when BMI was less than 30 kg/m^2^; however, when BMI was greater than 30 kg/m^2^, the lowering effect tended to become weaker with the increase of BMI (Figure 9). No significant cubic relationship was observed between effect size on IL-6 and other continuous confounding variables.

### Effect of Marine-Derived N-3 PUFAs from Dietary Intake (Fish Intake) on CRP, IL-6 and TNF-α in Subjects with Chronic Non-Autoimmune Disease

**Figure 10:**
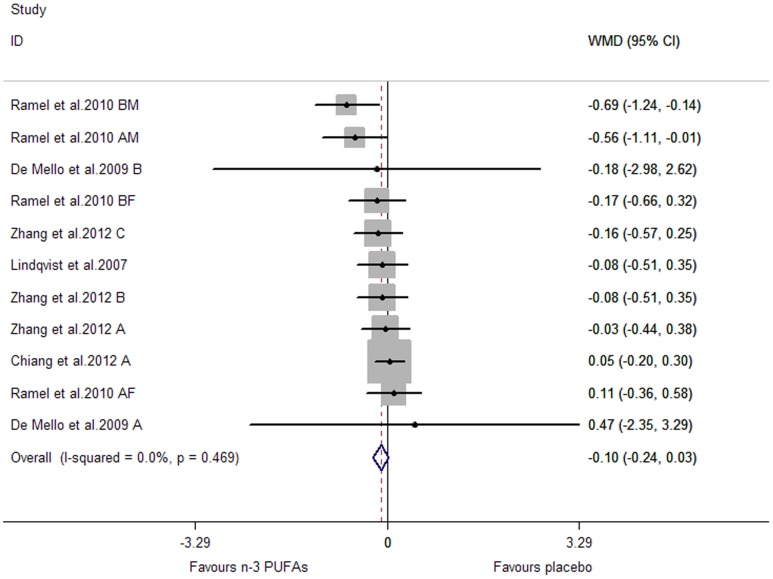
Pooled effect size of n-3 PUFAs from dietary intake on CRP in chronic non-autoimmune disease. WMD, weighted mean difference.

**Figure 11:**
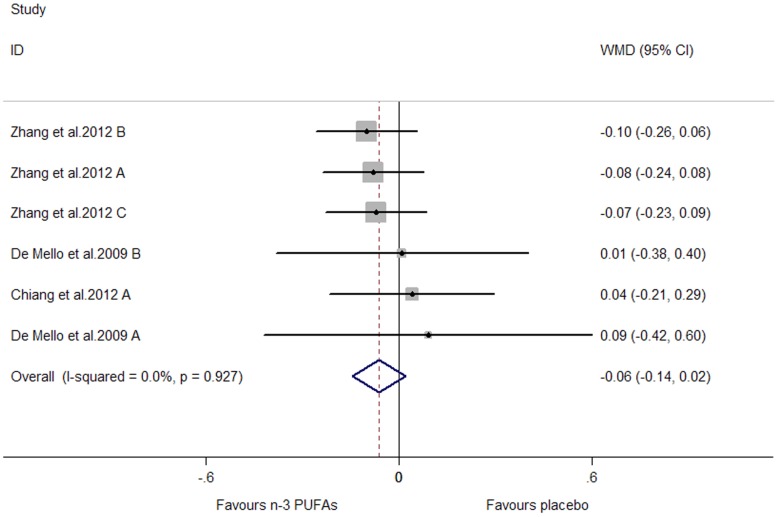
Pooled effect size of n-3 PUFAs from dietary intake on TNF-α in chronic non-autoimmune disease. WMD, weighted mean difference.

**Figure 12:**
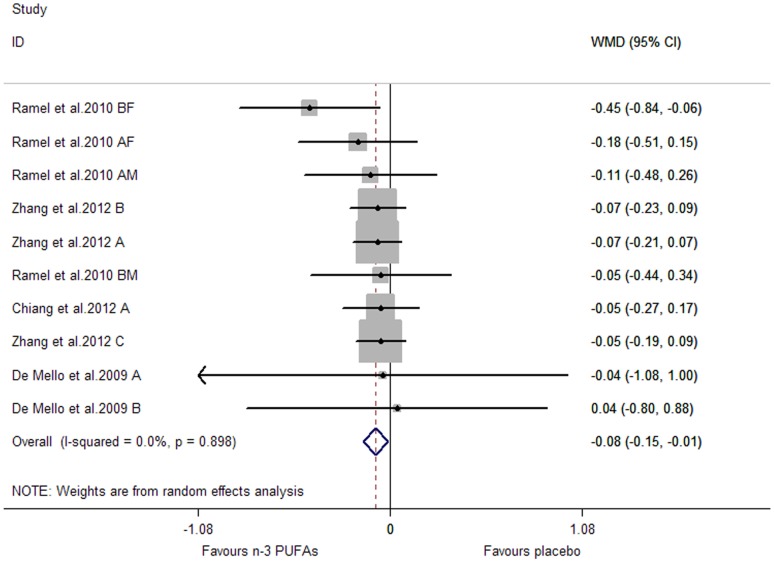
Pooled effect size of n-3 PUFAs from dietary intake on IL-6 in chronic non-autoimmune disease. WMD, weighted mean difference.

Subjects of all included studies had a high risk of or had been diagnosed with cardiovascular disease. The pooled effect sizes for CRP and TNF-α were not significant (P were 0.134 and 0.141, respectively) (Figure 10, Figure 11). A significant lowering effect on IL-6 was observed (WMD, −0.08; 95% CI, −0.15 to −0.01; P = 0.027) (Figure 12), and the overall percentage of change (in geometric mean) was −7.69% (95% CI, −13.93% to −1.00%). No heterogeneity was observed among the three groups of studies (I^2^ = 0.0%).

Subgroup analysis was conducted by the same methods used for marine-derived n-3 PUFAs supplementation (as were shown in Table 2). No significant subgroup difference was observed for CRP and IL-6 (P for subgroup difference >0.05). Subgroup analysis was not conducted for TNF-α, considering less than 10 studies (or comparisons) were included.

Meta-regression and restricted cubic spline analysis did not find any linear (P for coefficient >0.05) or cubic relationship (P for linearity >0.05) between effect size on CRP/IL-6 and continuous variables (as were mentioned in the results for marine-derived n-3 PUFAs supplementation). Meta-regression and restricted cubic spline analysis were not conducted for TNF-α, considering less than 10 studies (or comparisons) were included.

One study (two independent comparisons) assessing the effect on CRP had a risk of bias in two categories [95]. When we excluded it, the pooled effect size for CRP still remained non-significant.

### Effects of Marine-Derived N-3 PUFAs Supplementation on CRP, IL-6 and TNF-α in Subjects with Chronic Autoimmune Disease

**Figure 13:**
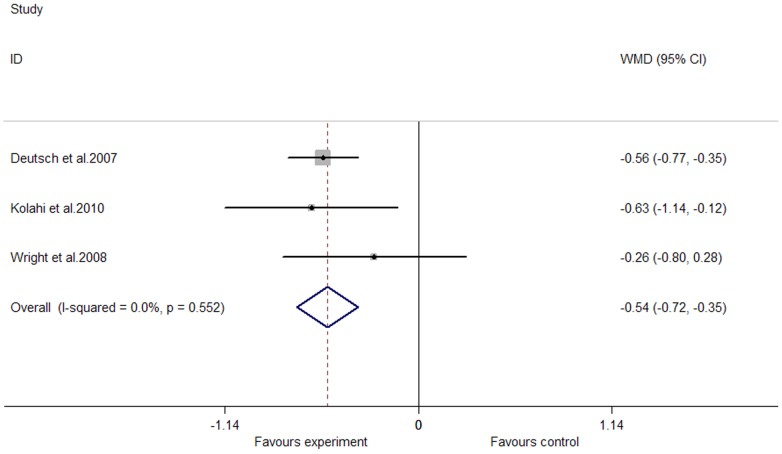
Pooled effect size of n-3 PUFAs supplementation on CRP in chronic autoimmune disease. WMD, weighted mean difference.

We observed a significant lowering effect of marine-derived n-3 PUFAs supplementation on fasting blood level of CRP; the pooled effect size was −0.54 (95% CI, −0.72 to −0.35; P = 0.000) (Figure 13), and the percentage of change (in geometric mean) was −41.73% (95% CI, −51.32% to −29.53%); no heterogeneity was observed in these studies (I^2^ = 0.0%); one study recruited both subjects with chronic non-autoimmune disease and subjects with chronic autoimmune disease [45], and after excluding this study the pooled effect size still remain significant. Only one study assessed the effect of marine-derived n-3 PUFAs supplementation on TNF-α [57], and a marginally significant lowering effect was observed (WMD, −0.28; 95% CI, −0.57 to 0.01; P = 0.061); the percentage of change (in geometric mean) was −24.42% (95% CI, −43.45% to 1.01%). No studies assessed the effect of marine-derived n-3 PUFAs supplementation on IL-6. Subgroup analysis, meta-regression and restricted cubic spline analysis were not conducted because the number of included studies was less than 10.

### Effects of Marine-Derived N-3 PUFAs Supplementation on CRP, IL-6 and TNF-α in Healthy Subjects

**Figure 14:**
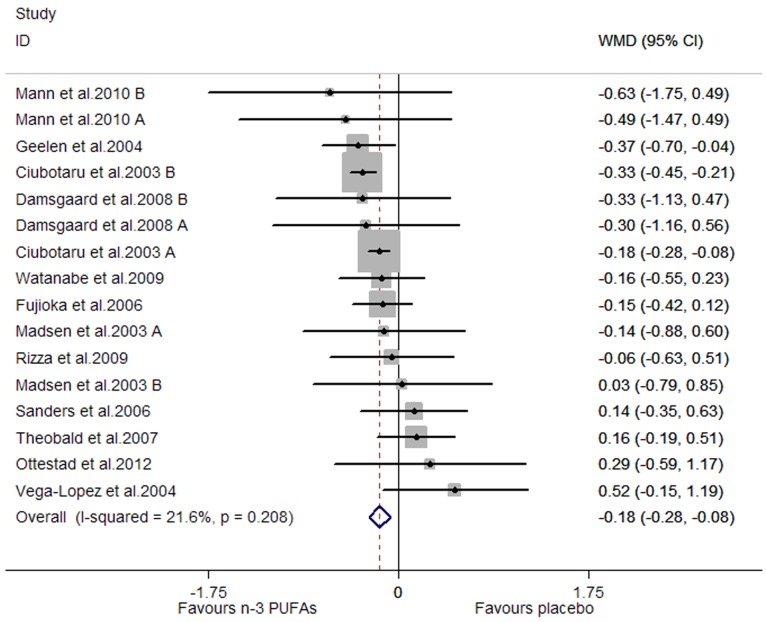
Pooled effect size of n-3 PUFAs supplementation on CRP in healthy subjects. WMD, weighted mean difference.

**Figure 15:**
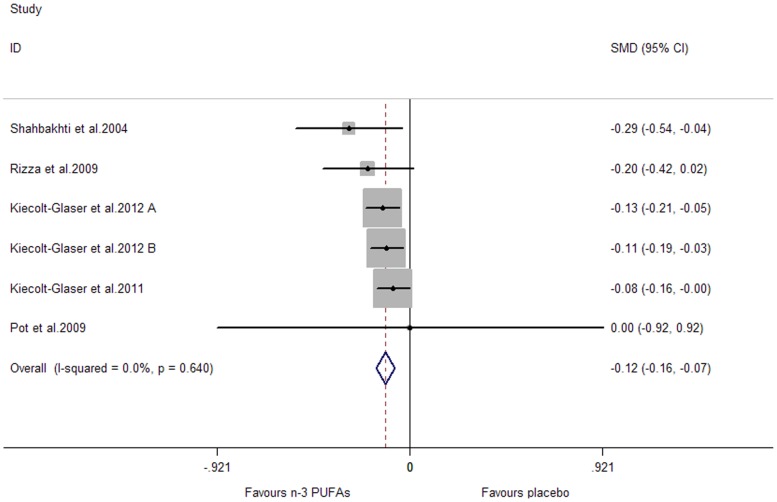
Pooled effect size of n-3 PUFAs supplementation on TNF-α in healthy subjects. WMD, weighted mean difference.

**Figure 16:**
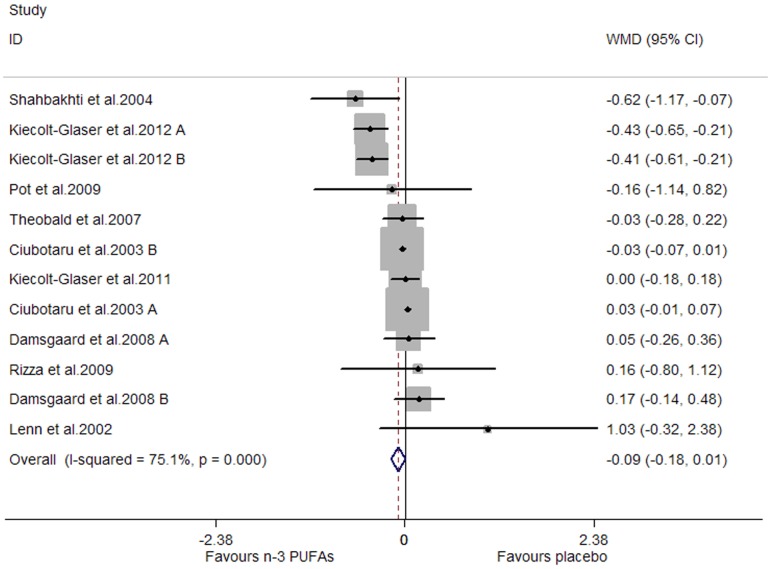
Pooled effect size of n-3 PUFAs supplementation on IL-6 in healthy subjects. WMD, weighted mean difference.

A significant lowering effect on CRP and TNF-α was observed, and the pooled effect sizes were -0.18 (95% CI, −0.28 to −0.08; P = 0.001) (Figure 14) and −0.12 (95% CI, −0.16 to −0.07; P = 0.000) (Figure 15), respectively; the percentages of changes (in geometric mean) were −16.47% (95% CI, −24.42% to −7.69%) and −11.31% (95% CI, −14.79% to −6.76%), respectively; no heterogeneity was observed for the two groups of studies (I^2^ values were 21.6% and 0.0%, respectively). A marginally significant lowering effect on IL-6 was observed (WMD, −0.09; 95% CI, −0.18 to 0.01; P = 0.065) (Figure 16), and the percentage of change (in geometric mean) was −8.61% (95% CI, −16.47% to 1.01%); significant heterogeneity was observed for studies assessing the effect on IL-6 (I^2^ = 75.1%).

Results of subgroup analysis for CRP and IL-6 were shown in Table 3. No significant subgroup difference was observed for CRP (P for subgroup difference >0.05). Length of duration had a significant influence on effect size for IL-6: significant lowering effect was observed only when duration was longer than median, and the pooled effect size for this subgroup was −0.24 (95% CI, −0.44 to −0.03; P = 0.022) (P for subgroup difference was 0.037); the variation of duration (as categorical variable) could help explain 45.44% heterogeneity. Subgroup analysis was not conducted for TNF-α, considering the number of included studies (comparisons) was less than 10.

**Table 3:** The effect of marine-derived n-3 PUFAs supplementation on CRP, IL-6 in healthy subjects (data were log-transformed before analysis).

	CRP				IL-6				
Subgroup analysis	N	WMD (95% CI)	I^2^ (%)	P	N	WMD (95% CI)	I^2^ (%)	P	
Overall	16	−0.18 (−0.28, −0.08)	21.6	0.001	12	−0.09 (−0.18, 0.01)	75.1	0.065	
Study design									
Parallel	12	−0.20 (−0.31, −0.09)	24.8	0.000	9	−0.13 (−0.24, −0.02)	81.2	0.021	
Crossover	2	−0.01 (−0.30, 0.32)	29.3	0.941	1	−0.03 (−0.28, 0.22)	NA	0.817	
Factorial	2	−0.32 (−0.90, 0.27)	0.0	0.292	2	0.11 (−0.11, 0.33)	0.0	0.331	
Major fatty acid in placebo									
Linoleic acid	2	−0.25 (−0.40, −0.10)	72.9	0.001	2	0.00 (−0.06, 0.06)	77.8	1.000	
Oleic acid	13	−0.12 (−0.26, 0.02)	0.0	0.084	6	−0.02 (−0.21, 0.17)	21.7	0.845	
Others or not reported	1	0.52 (−0.15, 1.19)	NA	0.126	4	−0.22 (−0.52, 0.09)	81.7	0.160	
Duration (week)									
< median	7	−0.22 (−0.34, −0.11)	26.7	0.000	5	**0.01 (**−**0.05, 0.07)**	49.6	0.760	
≥ median	9	−0.09 (−0.26, 0.07)	8.1	0.266	7	−**0.24 (**−**0.44,** −**0.03)**	66.7	0.022	
Daily dose of total n-3 PUFAs (g)									
< median	8	−0.12 (−0.29, 0.05)	36.2	0.162	6	−0.08 (−0.33, 0.17)	76.3	0.543	
≥ median	8	−0.28 (−0.38, −0.17)	0.0	0.000	6	−0.10 (−0.26, 0.07)	74.2	0.236	
Daily dose of EPA (g)									
< median	8	−0.11 (−0.27, 0.05)	38.5	0.192	5	−0.11 (−0.36, 0.14)	78.9	0.369	
≥ median	8	−0.29 (−0.39, −0.18)	0.0	0.000	6	−0.10 (−0.26, 0.07)	74.2	0.236	
Not reported	0	NA	NA	NA	1	1.03 (−0.32, 2.38)	NA	0.136	
Daily dose of DHA (g)									
< median	8	−0.17 (−0.26, −0.09)	0.0	0.000	5	−0.24 (−0.48, 0.00)	89.8	0.052	
≥ median	8	−0.09 (−0.33, 0.15)	38.0	0.478	6	−0.03 (−0.06, 0.01)	0.0	0.185	
Not reported	0	NA	NA	NA	1	1.03 (−0.32, 2.38)	NA	0.136	
Mean age (year)									
< median	8	0.00 (−0.25, 0.25)	0.0	0.995	6	0.01 (−0.20, 0.22)	40.5	0.913	
≥ median	8	−0.21 (−0.31, −0.10)	29.8	0.000	6	−0.13 (−0.24, −0.02)	85.9	0.022	
Sex ratio (male/total subjects)									
<0.5	6	−0.24 (−0.33, −0.14)	19.2	0.000	5	−0.17 (−0.30, −0.05)	90.0	0.007	
≥0.5	9	−0.10 (−0.27, 0.06)	0.0	0.219	7	0.03 (−0.09, 0.15)	0.0	0.584	
Not reported	1	0.52 (−0.15, 1.19)	NA	0.126	0	NA	NA	NA	
Baseline (log-transformed)									
< median	8	−0.07 (−0.24, 0.10)	0.0	0.410	5	0.00 (−0.07, 0.07)	62.4	0.995	
≥ median	8	−0.21 (−0.35, −0.07)	44.5	0.004	6	−0.23 (−0.47, 0.01)	56.9	0.060	
Not reported	0	NA	NA	NA	1	0.00 (−0.18, 0.18)	NA	1.000	

N, number of included studies (or comparisons); NA, not associated with this item.

Unit for CRP: mg/L; unit for IL-6: pg/mL.

Data expressed in bold indicated that subgroup difference was significant (P for subgroup difference <0.05), and subgroup “not reported” was not included in the significance test for subgroup difference.

**Figure 17:**
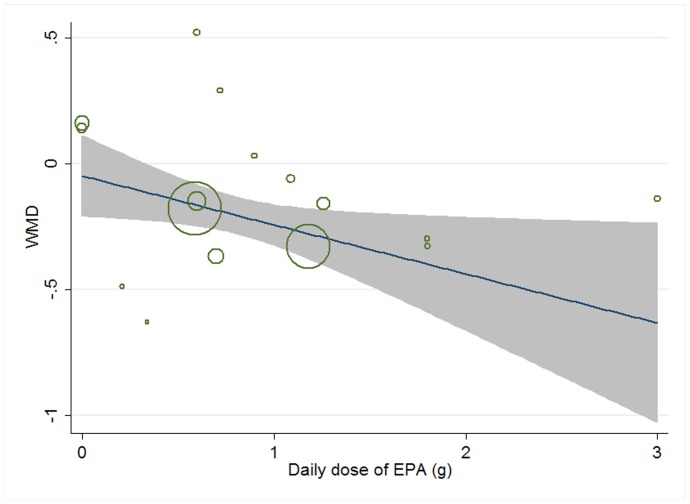
Meta-regression for daily dose of EPA and effect size (n-3 PUFAs supplementation on CRP in healthy subjects). WMD, weighted mean difference.

**Figure 18:**
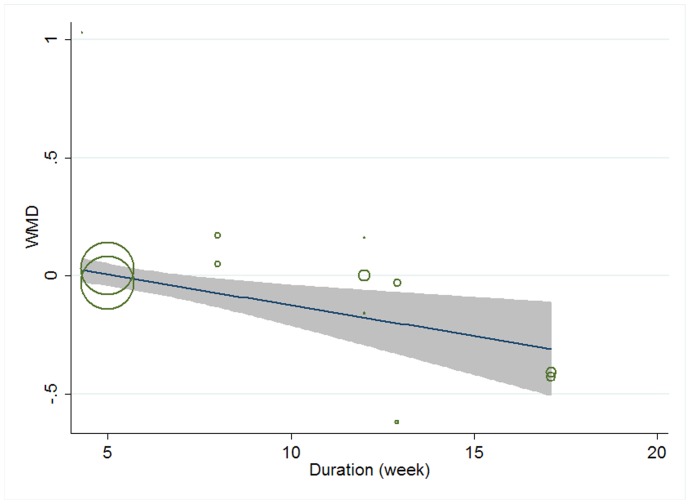
Meta-regression for duration and effect size (n-3 PUFAs supplementation on IL-6 in healthy subjects). WMD, weighted mean difference.

**Figure 19:**
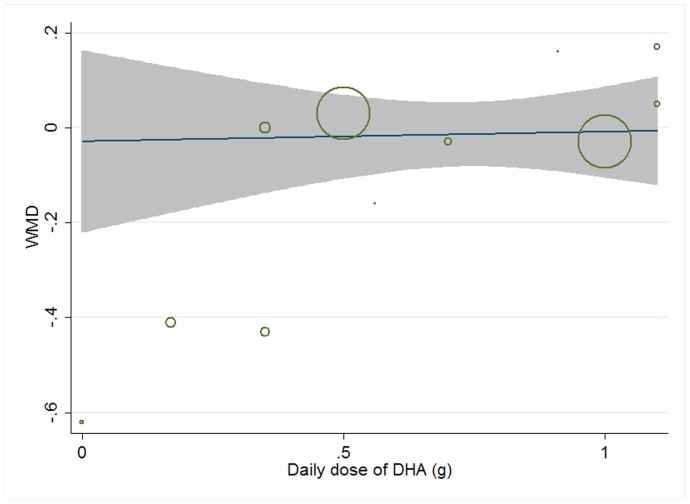
Meta-regression for daily dose of DHA and effect size (n-3 PUFAs supplementation on IL-6 in healthy subjects). WMD, weighted mean difference.

Meta-regression was conducted to explore potential linear relationship between effect size and continuous confounding factors (baseline, duration, age, sex ratio (male/total subjects), and daily dose of EPA, DHA, and total n-3 PUFAs). A significant linear relationship was observed between effect size on CRP and daily dose of EPA, and the coefficient was −0.195 (95% CI, −0.374 to −0.016; P = 0.035) (Figure 17), indicating that higher doses of EPA could lead to a greater lowering effect, and the percentage of change (in geometric mean) with one unit increase of EPA dose (g/d) was −17.72% (95% CI, −31.20% to −1.59%); no significant linear relationship was observed between effect size on CRP and other continuous confounding variables. A significant linear relationship was observed between effect size on IL-6 and duration as well as daily dose of DHA, and the correlations were −0.032 (95% CI, −0.054 to −0.011; P = 0.007) (Figure 18) and 0.450 (95% CI, 0.055 to 0.845; P = 0.030) (Figure 19), respectively, indicating that longer duration and lower daily dose of DHA could lead to a greater lowering effect; the percentages of changes (in geometric mean) with one unit increase of duration (week) and DHA dose (g/d) were −3.15% (95% CI, −5.26% to −1.09%) and 56.83% (95% CI, 5.65% to 132.80%), respectively; joint test for the two covariates above (as continuous variables) showed that their variation could help explain 57.69% heterogeneity; no significant linear relationship was observed between effect size on IL-6 and other continuous confounding variables. The significant linear relationship between duration and effect size demonstrated that marine-derived n-3 PUFAs supplementation also had a significant lowering effect on IL-6 in healthy subjects although the pooled effect size was only marginally significant. Meta-regression was not conducted for TNF-α, considering the number of included studies (comparisons) was less than 10.

**Figure 20:**
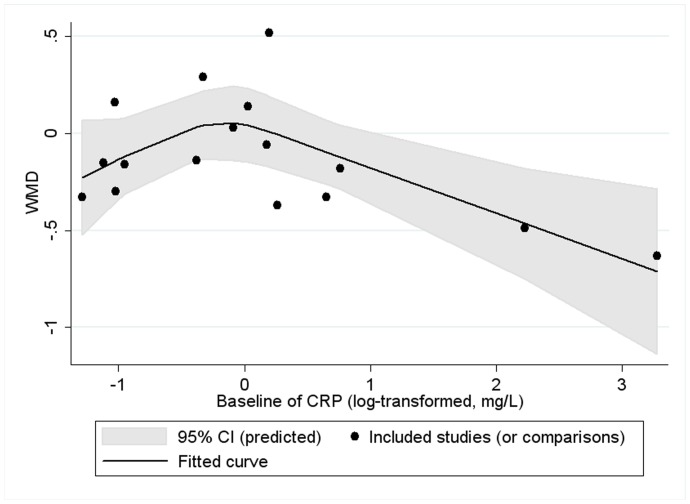
Cubic relationship between baseline of CRP and effect size (n-3 PUFAs supplementation on CRP in healthy subjects). WMD, weighted mean difference.

Restricted cubic spline analysis for IL-6 showed no significant cubic relationship between effect size on the two inflammatory factors and continuous confounding factors (as were mentioned in the paragraph above) (P for linearity >0.05). We found a significant cubic relationship between baseline of CRP and effect size on CRP (P for linearity = 0.021) (Figure 20): the lowering effect was slightly weakened with the increase of baseline (log-transformed, mg/L) when baseline (log-transformed) was less than 0; however, when baseline (log-transformed, mg/L) was greater than 0, the lowering effect became significantly greater with the increase of baseline. Restricted cubic spline analysis was not conducted for TNF-α, considering the number of included studies (comparisons) was less than 10.

### Mean Changes of Phospholipid N-3 PUFAs in Plasma/Serum and the Effect Size of Marine-Derived N-3 PUFAs

Data of mean changes for phospholipid EPA, DHA and total n-3 PUFAs were shown in Table S1. Subgroup, meta-regression and restricted cubic spline analyses were conducted to explore the association between changes of phospholipid EPA, DHA and total n-3 PUFAs in plasma/serum and the effect size of marine-derived n-3 PUFAs. We chose changes of phospholipid EPA, DHA and total n-3 PUFAs in plasma/serum because only a small part of included studies reported changes of n-3 PUFAs in tissue and most of them presented n-3 PUFAs changes in tissue as changes of phospholipid EPA, DHA and total n-3 PUFAs in plasma/serum. Considering that the three types of analyses cannot obtain enough power unless the number of included studies was at least 10, we included studies used marine-derived n-3 PUFA from different sources (supplementation or dietary intake) and studies recruited subjects with different healthy status (subjects with chronic non-autoimmune disease, chronic auto-immune disease and healthy subjects) into these analyses simultaneously. Subgroup, meta-regression and restricted cubic spline analyses all showed no significant results for CRP and IL-6. The three types of analysis were not conducted for TNF-α, because less than 10 studies reported the association between the effect size on TNF-α and changes of phospholipid EPA, DHA or total n-3 PUFAs in plasma/serum.

### Publication Bias

The funnel plot for studies assessing the effect of marine-derived n-3 PUFAs supplementation on IL-6 in subjects with chronic non-autoimmune disease showed a slight asymmetry, indicating that considerable publication bias existed (Figure S3); trim-and-fill test suggested that 7 studies needed to be trimmed to make the funnel symmetric, and after the addition of 7 studies, the pooled effect size on IL-6 became −0.35 (95% CI, −0.49 to −0.20) and still remained significant (p = 0.000); the corresponding percentage of change (in geometric mean) for IL-6 was −29.53% (95% CI, −38.74% to −18.13%). Other funnel plots for meta-analyses including at least 10 studies did not show any significant asymmetry, indicating that no significant publication bias existed among these groups of studies (Figure S3–S9). Funnel plot analysis for meta-analysis including less than 10 studies was not conducted considering that this method cannot obtain enough power when the number of included studies is less than 10 (as mentioned in Statistical Methods).

## Discussion

The present meta-analysis provided consistent evidence that marine-derived n-3 PUFAs supplementation had a significant lowering effect on fasting blood levels of CRP, IL-6 and TNF-α in subjects with chronic non-autoimmune disease and healthy subjects. Marine-derived n-3 PUFAs supplementation had a significant lowering effect on fasting blood level of CRP and a marginally significant lowering effect on TNF-α in subjects with chronic autoimmune disease; no included studies assessed the effect on IL-6 in subjects with chronic autoimmune disease. The effect of marine-derived n-3 PUFAs from dietary intake on CRP, IL-6 and TNF-α was only assessed in subjects with chronic non-autoimmune disease, and significant lowering effect was only observed on IL-6, but not on CRP and TNF-α.

The lowering effect of n-3 PUFAs on CRP, IL-6 and TNF-α has a biological basis. The mechanism may involve both nuclear factor кB (NF-кB) and peroxisome proliferator agonist receptors (PPAR). N-3 PUFAs has been found to be a natural ligand of PPAR [99]. PPAR was a ligand-activated transcription factor. The heterodimeric complex comprised of PPAR and its ligand can bind to peroxisome proliferator response elements on DNA to regulate gene expression [100]. A previous study showed that PPAR inhibited the activation of NF-кB [101], which can initiate genes encoding for inflammatory factors, such as TNF-α and IL-6 [102]. Moreover, TNF-α also plays a role in the activation of NF-кB by leading to phosphorylation of IкBa, an inhibitor of NF-кB [103]. Phosphorylation of IкBa can cause the dissociation of IкBa from NF-кB, and further the proteolysis of IкBa due to the exposure of a previously masked proteolytic cleavage site [103]. Therefore, the down-regulation effect of n-3 PUFAs on TNF-α by PPAR and NF-кB signal pathways may in turn interfere with the activation of NF-кB, leading to a further reduction in the levels of TNF-α and IL-6. In addition, TNF-α and IL-6 play an important role in acute phase inflammatory response, including induction of acute phase reactant protein production (e.g., CRP) [10]. In addition, one previous study showed that high oxidative stress can led to the activation of NF-кB [104]. A review by Mori et al. found that EPA and DHA had a significant lowering effect on oxidative stress [105]. As suggested above, NF-кB can initiate genes encoding for inflammatory factors, such as TNF-α and IL-6, and thus increase the level of CRP. Therefore, the antioxidative effect of n-3 PUFAs may also help explain its lowering effect on TNF-α, IL-6 and CRP.

By subgroup analysis, we found that fatty acid compositions of placebo had a significant influence on the effect size of marine-derived n-3 PUFAs supplementation on CRP in subjects with chronic non-autoimmune disease, the significant lowering effect of marine-derived n-3 PUFAs was observed when oils mainly comprised of linoleic acid (n-6 fatty acid) rather than oleic acid (monosaturated fatty acid) were used as placebo (p for subgroup difference <0.05). A similar result was also observed in healthy subjects: although the subgroup difference was not significant (p for subgroup difference >0.05), significant result was observed only in subgroup using oils mainly comprised of linoleic acid (n-6 fatty acid) rather than oleic acid (monounsaturated fatty acid). Reasons for this difference induced by placebo might be explained by the role different fatty acid play in the immune system. Previous studies have demonstrated that n-6 polyunsaturated fatty acid play an opposing role with n-3 polyunsaturated fatty acid in the process of inflammation [106], [107]: n-6 polyunsaturated fatty acid can initiate the activation of NF-кB by arachidonic acid, an intermediate product of n-6 fatty acid during metabolism, and thus increase the level of TNF-α, IL-6 and CRP as suggested in the paragraph above. However, oleic acid has an anti-inflammatory property by suppressing lymphocyte proliferation, inhibiting cytokine production and reducing activity of NK cells [108]. The opposing role of linoleic acid (n-6 polyunsaturated fatty acid) and oleic acid (monounsaturated fatty acid) in inflammation may help explain the difference in effect size caused by placebo.

By meta-regression, we observed a significant negative linear relationship between duration and effect size of marine-derived n-3 PUFAs supplementation on IL-6 and TNF-α in subjects with chronic non-autoimmune disease, indicating that longer duration of use could lead to a greater lowering effect. Similar linear relationship was also observed between duration and effect size of marine-derived n-3 PUFAs supplementation on IL-6 in healthy subjects. The relationship between duration and effect size was easy to understand if we take into consideration that changing fatty acid composition of cell membrane and tissues by n-3 PUFAs intake needs a relatively long term. One previous controlled study by Katan et al. assessed the effect of fish oil intake on changes of fatty acid composition in serum cholesteryl esters, erythrocyte membranes and adipose tissue during an 18-month follow-up [109]. The results showed that EPA plateaued only after 4–8 weeks in serum cholesteryl esters. However, the steady period for EPA in erythrocyte membrane and gluteal fat tissue did not come until supplementation consisted for 6 months and 12 months, respectively, and the content of EPA in abdominal fat tissue still remain a trend to increase after supplementation for 12 months. Therefore, the result observed in our present study that the lowering effect size of marine-derived n-3 PUFAs supplementation on inflammatory factors is greater with the extension of duration when duration was shorter than 12 months, is consistent with the study by Katan et al. [109] and is plausible.

We observed a significant cubic relationship between BMI of subjects and the effect size of marine-derived n-3 PUFAs supplementation on fasting blood level of IL-6 in subjects with chronic non-autoimmune disease in studies with a parallel group design. From the fitted curve, we found that the lowering effect of marine-derived n-3 PUFAs supplementation on IL-6 became weakened when BMI was greater than 30 kg/m^2^. Although cubic relationships between BMI and the effect sizes of marine-derived n-3 PUFAs supplementation on the level of CRP and TNF-α in subjects with chronic non-autoimmune disease were not significant, subgroup analysis found a similar result: marine-derived n-3 PUFAs supplementation had a significant lowering effect on CRP and TNF-α in subjects with a BMI less than 30 kg/m^2^ but not in subjects with a BMI ≥30 kg/m^2^, and the subgroup difference was significant. One possible reason for the lack of correlation in obese subjects may be explained by the association between fatty acid composition in tissue and obesity. A previous study found that the total n-6 PUFAs and arachidonic acid in adipose tissues all increased with BMI in obese subjects [110]. As we know, arachidonic acid can produce several pro-inflammatory eicosanoids through cyclooxygenase (COX) and 5-lipoxygenase (5-LOX) pathways. One important mechanism for the anti-inflammatory effect of n-3 PUFAs can be attributed to its competing role with arachidonic acid in eicosanoids metabolism: n-3 PUFAs can inhibit the oxidation of arachidonic acid by cyclooxygenase (COX) enzymes, and thus reducing the production of pro-inflammatory eicosanoids, such as prostaglandin E2 (PGE2) and thromboxane A2 (TXA2); n-3 PUFAs are also substrates for COX and 5-LOX; n-3 PUFAs can be incorporated into membrane phospholipids at the expense of arachidonic acid, and thus reduce the production of eicosanoids from arachidonic acid [111]. Arachidonic acid-derived eicosanoids also play an important role in regulating the production of several cytokines, such as TNF-α and IL-6 [112] and further regulating the production of other inflammatory markers such as CRP. Therefore, higher n-6 PUFAs and arachidonic acid level in tissue of obese subjects may needs a higher dosage of n-3 PUFAs than non-obese subjects to reduce the production of arachidonic acid-derived eicosanoids, CRP, IL-6 and TNF-α. This was demonstrated by one study which indicated that a high dose of n-3 PUFA (4.2g/d) supplementation significantly reduced CRP and IL-6 levels [38]. However, most included studies in our present meta-analysis which recruited obese subjects used a much lower dose than that. This may be the reason why we did not observe a lowering effect of marine-derived n-3 PUFAs on CRP, IL-6 and TNF-α in obese subjects. In addition, other medicine use and difference in compliance may also be the potential factors that led to the non-significant results in obese subjects. However, most included studies recruiting obese subjects excluded subjects taking medicine with a potential influence on inflammatory response and reported a high degree of compliance assessed by capsules count or determination of fatty acid changes in tissue. Therefore, other medicine use and difference in compliance seems not to be the reasons for the difference of effect size caused by BMI in our present meta-analysis.

A significant negative linear relationship between age and the effect size of marine-derived n-3 PUFAs supplementation on fasting blood level of IL-6 was observed in subjects with chronic non-autoimmune disease, indicating that the lowering effect was stronger in older subjects. Subgroup analysis also found a consistent result. One previous study by Rodrigo et al. also found that older subjects showed a better antioxidant response to n-3 PUFAs supplementation [113]. As suggested in the second paragraph of this discussion, one possible mechanism for the anti-inflammatory effect of n-3 PUFAs might be attributed to its antioxidant effect. Therefore, our result concerning the effect of age on the association between marine-derived n-3 PUFAs supplementation and IL-6 was consistent with the study by Rodrigo et al. [113]. One study by Rees et al. found that older subjects incorporated long-chain n-3 PUFAs into plasma and mononuclear cells more readily than younger ones [114]. Similar results were also found in the study by Meydani et al. [115]. These results were consistent with one cross-sectional study which found that long-chain n-3 PUFAs in adipose tissue increased with age [116]. This may be one reason why the lowering effect of marine-derived n-3 PUFAs supplementation on IL-6 was more effective in older subjects. In addition, one study by Cartier et al. found that CRP, IL-6 and TNF-α level increased with age in men [117]. Our present study showed that higher baseline level was associated with a greater lowering effect (Table 2, Figure 7 and Figure 20). This may also help explain the difference in effect size caused by age.

We also observed a significant negative linear relationship between daily dose of EPA and the effect size on fasting blood level of CRP in healthy subjects, indicating that higher dose of EPA might lead to a greater lowering effect on CRP. But similar relationship was not observed for daily dose of total n-3 PUFAs or DHA. This provided some evidence that EPA might be at least more effective than DHA on lowering fasting blood level CRP. We also observed a significant positive linear relationship between daily dose of DHA and the effect size on fasting blood level of IL-6 in healthy subjects. This result was consistent with the negative relationship between daily dose of EPA and the effect size on fasting blood level of CRP if we take into consideration that marine-derived n-3 PUFA supplementation (mainly fish oil) is mainly comprised of EPA and DHA, and that higher dose of DHA might lead to a lower proportion of EPA. One previous studies in mice showed that the lowering effect on cytokines production by thioglycollate-induced macrophages were only significant in the group fed by diet with the highest EPA/DHA ratio [118]. These results came to a consistence with those of our present meta-analysis. The mechanism behind this phenomenon may involve the competing role of n-3 PUFAs (mainly EPA) with arachidonic acid in eicosanoids metabolism which has been described in detail in the fifth paragraph of discussion. Briefly, EPA reduces the production of arachidonic acid-derived eicosanoids by inhibiting the oxidation of arachidonic acid by COX, acting as competing substrates of COX and 5-LOX, and incorporating into cell membrane phospholipids at the expense of arachidonic acid, and thus reduces the production of cytokines (such as IL-6 and TNF-α) and other inflammatory factors (such as CRP). Although previous studies have shown that DHA can be retroconverted to EPA, the retroconversion ratios were low (about 7.4% and 11.4% in young adult omnivores and vegetarians, respectively) [119]. In addition, one previous study in mice showed that diet high in EPA rather than diet high in DHA significantly increased IL-10 production by macrophages and lymphocytes; two diets all led to a similar increase of DHA in tissue, but significant EPA increase was only observed after EPA diet intake [120]. As we know, one important function of IL-10 is to inhibit cytokine production [121]. Interestingly, one study found that DHA can even induce T helper cell type 1-like immune response by increase the ratio of interferon-γ to IL-10 but not for EPA [122]. These points above might help explain why EPA and DHA showed different effect in regulating the process of inflammatory response.

However, our present meta-analysis showed that changes of phospholipid EPA, DHA and total n-3 PUFAs in plasma/serum did not show any significant influence on their effect size concerning CRP, IL-6 and TNF-α. As mentioned in the fourth paragraph of discussion, EPA in serum cholesteryl esters, erythrocyte membrane and gluteal fat tissue plateaued after 4–8 weeks, 6 months and 12 months, respectively, and the content of EPA in abdominal fat tissue still remain a trend to increase after supplementation for 12 months [109]. However, the duration of most included studies in our present meta-analysis ranged from 6 weeks to 4 months. Therefore, the change of phospholipid fatty acid in plasma/serum cannot absolutely represent that in cell membrane or fat tissue, that is, studies with a longer duration may have a similar fatty acid fatty acid in plasma/serum but differ in that of cell membrane or fat tissue. However, the effects of these changes on effect size were not analyzed in the present meta-analysis because only few included studies reported fatty acid changes in cell membrane or fat tissue. In addition, one limit for the analyses (subgroup, meta-regression and restricted cubic analyses) on the association between changes of phospholipid fatty acid in plasma/serum and effect size was that we included studies using marine-derived n-3 PUFAs from different sources (supplementation or dietary intake) and studies recruited subjects with different healthy status (subjects with chronic disease or healthy subjects) into these analyses simultaneously. We did so because these analyses cannot obtain enough power unless the number of included studies was at least 10. However, this makes sources of marine-derived n-3 PUFAs and healthy status of subjects become potential confounding factors which might influence the final results.

One previous study reviewed the effect of marine-derived n-3 PUFAs on inflammatory markers in randomized controlled trials, and found that only few studies showed a significant lowering effect on CRP, IL-6 and TNF-α in healthy subjects, and that the lowering effect of marine-derived n-3 PUFAs was significant on CRP and IL-6 in subjects with a presence or high risk of cardiovascular disease [23]. The effect on TNF-α in subjects with a presence or high risk of cardiovascular disease was significant in only a few studies [23]. Another review also found that the lowering effect of marine-derived n-3 PUFAs on CRP was associated with the decreased level of IL-6 [24]. This was consistent with the results in our present study. However, because the results from different studies were contradictory, both reviews did not draw a firm conclusion about the association between n-3 PUFAs and inflammatory factors. Xin et al. conducted a meta-analysis for the association between fish oil supplementation and inflammatory markers in subjects with chronic heart failure [25]. The study found a significant lowering effect of fish oil on circulating level of IL-6 and TNF-α, and subgroup analysis indicated that the lowering effect on IL-6 and TNF-α was greater in studies with a longer duration (>4 months) than studies with a shorter duration (≤4 months) [25]. This result was consistent with our present findings concerning the effect of marine-derived n-3 PUFAs supplementation in subjects with chronic non-autoimmune diseases. However, the meta-analysis by Xin et al. founds a significant association between circulating level of CRP and fish oil supplementation. There were several different points in study design between the study by Xin et al. and our present meta-analysis. Firstly, our present meta-analysis combined the results from studies assessing the effect of marine-derived n-3 PUFAs supplementations in subjects with all kinds of chronic non-autoimmune diseases into a single meta-analysis, and thus incorporated much more subjects than the meta-analysis by Xin et al., and subgroup analysis according to types of diseases did not show any significant subgroup difference. Secondly, both the meta-analysis by Xin et al. and our present meta-analysis included skewed data (such as results expressed as log-transformed data or median). The study by Xin et al. transformed skewed data into mean ± SD, and combined these data on raw scale. However, considering that meta-analysis is based on the assumption that data are normally distributed, we transformed all of the data into log-transformed scale, which can substantially reduce skew, and then conducted the data analysis [27], [29]. By data transformation, the bias caused by skew of data on raw scale was eliminated in our present study. Thirdly, of the 7 studies [20], [71], [77], [78], [123]–[125] included in the meta-analysis by Xin et al., 3 studies were not included into our present study [123]–[125]. Reasons for exclusion were as follows. One study supplemented subjects with fish oil and assessed changes of cytokines production by *ex vivo* mononuclear cells before and after supplementation [124]. This study was excluded considering there was considerable heterogeneity between cytokines production by *ex vivo* mononuclear cells and fasting blood levels of cytokines *in vivo*. Another study used a crossover design to assess the effect of fish oil on fasting blood level of CRP, IL-6 and TNF-α [125]. However, it did not report a wash-out period. Considering the carry-over effect might be significant without a wash-out period, this study was also excluded. The third study was excluded because the reported data cannot be transformed into log-scale by the methods suggested in “Statistical Methods” and log-transformed data was still unavailable after contacting the author [123].

Several strengths can be observed in the present study. Firstly, we conducted meta-analysis based on log-transformed data, and this transformation eliminated potential bias caused by skew of data in some included studies. Secondly, our present study showed that the effect of marine-derived n-3 PUFAs supplementation had a lowering effect on all the three inflammatory factors (CRP, IL-6 and TNF-α). Considering the up-regulation effect of TNF-α on IL-6 and up-regulation effect of IL-6 on CRP (discussed in the second paragraph of discussion), the similarity of results concerning CRP, IL-6 and TNF-α strengthened the persuasion of our study. In addition, the large number of included studies and large sample size also make our results more plausible.

Our study also had several limitations. Firstly, a risk of bias was observed in several studies included in the present meta-analysis. But results from our sensitivity analysis showed that these biases did not have a significant influence on the pooled effect size. Secondly, a significant heterogeneity was observed in the three meta-analyses assessing the effect of marine-derived n-3 PUFAs supplementation on CRP, IL-6 and TNF-α in subjects with chronic non-autoimmune disease as well as the meta-analysis evaluating the effect of marine-derived n-3 PUFAs supplementation on IL-6 in healthy subjects. However, the sources of heterogeneity and their influence on the effect size were well explained by subgroup meta-analysis, meta-regression and restricted cubic spline analysis.

Our findings provide a scientific guide for nutritional therapy of inflammation-related chronic diseases. Consecutive long-term supplementation of marine-derived n-3 PUFAs is recommended. Our present studies also suggest that marine-derived n-3 PUFAs supplementation can effectively prevent inflammation-related chronic diseases considering its significant lowering effect on CRP, IL-6 and TNF-α in healthy subjects. The anti-inflammatory effect tends to be the most effective in non-obese subjects.

In conclusion, marine-derived n-3 PUFAs supplementation has a significant lowering effect on fasting blood level of TNF-α, IL-6 and CRP.

## Supporting Information

Figure S1 Judgements about each risk of bias item presented as percentages across all included studies.
(PDF)

Figure S2 Judgements about each risk of bias item for each included study. Red ball, high risk of bias; yellow ball, unclear risk of bias; green ball, no risk of bias.
(PDF)

Figure S3 Funnel plot for publication bias (n-3 PUFAs supplementation and IL-6 in chronic non-autoimmune disease). SE, standard error; WMD, weighted mean difference.
(TIF)

Figure S4 Funnel plot for publication bias (n-3 PUFAs supplementation and CRP in chronic non-autoimmune disease). SE, standard error; WMD, weighted mean difference.
(TIF)

Figure S5 Funnel plot for publication bias (n-3 PUFAs supplementation and TNF-α in chronic non-autoimmune disease). SE, standard error; WMD, weighted mean difference.
(TIF)

Figure S6 Funnel plot for publication bias (n-3 PUFAs from dietary intake and CRP in chronic non-autoimmune disease). SE, standard error; WMD, weighted mean difference.
(TIF)

Figure S7 Funnel plot for publication bias (n-3 PUFAs from dietary intake and IL-6 in chronic non-autoimmune disease). SE, standard error; WMD, weighted mean difference.
(TIF)

Figure S8 Funnel plot for publication bias (n-3 PUFAs supplementation and CRP in healthy subjects). SE, standard error; WMD, weighted mean difference.
(TIF)

Figure S9 Funnel plot for publication bias (n-3 PUFAs supplementation and IL-6 in healthy subjects). SE, standard error; WMD, weighted mean difference.
(TIF)

Table S1 Baseline of inflammatory markers (log-transformed) and mean changes of phospholipid n-3 PUFAs in plasma/serum.
(DOC)

Checklist S1 PRISMA checklist.
(DOC)

Text S1 Protocol for meta-analysis to assess the effect of marine-derived n-3 polyunsaturated fatty acids on CRP, IL-6 and TNF-α.
(DOC)
