# Supplementary figures and images for: Effect of Marine-Derived n-3 Polyunsaturated Fatty Acids on C-Reactive Protein, Interleukin 6 and Tumor Necrosis Factor α: A Meta-Analysis
Source: PLoS One. 2014 Feb 5;9(2):e88103. doi: 10.1371/journal.pone.0088103 (PMC3914936; doi:10.1371/journal.pone.0088103)

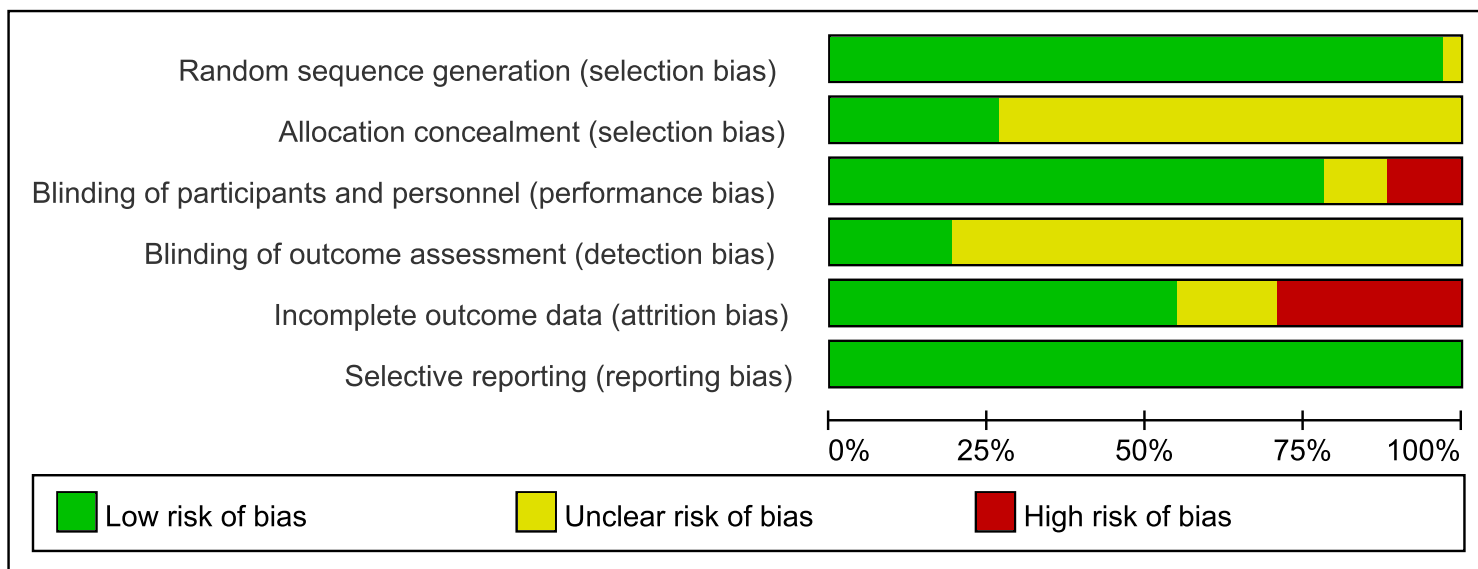

Supplement: Figure S1 — Judgements about each risk of bias item presented as percentages across all included studies. (PDF) [file pone.0088103.s001.pdf]

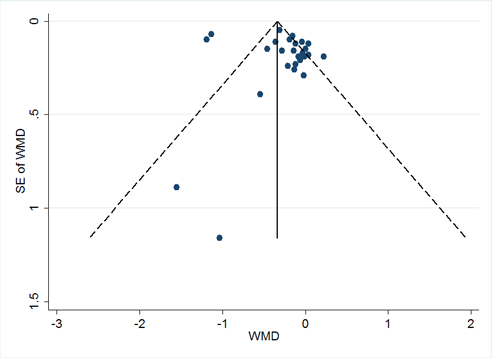

Supplement: Figure S3 — Funnel plot for publication bias (n-3 PUFAs supplementation and IL-6 in chronic non-autoimmune disease). SE, standard error; WMD, weighted mean difference. (TIF) [file pone.0088103.s003.tif]

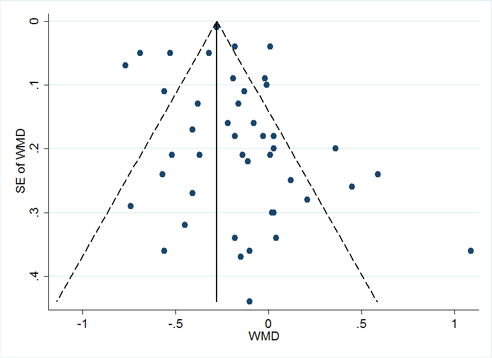

Supplement: Figure S4 — Funnel plot for publication bias (n-3 PUFAs supplementation and CRP in chronic non-autoimmune disease). SE, standard error; WMD, weighted mean difference. (TIF) [file pone.0088103.s004.tif]

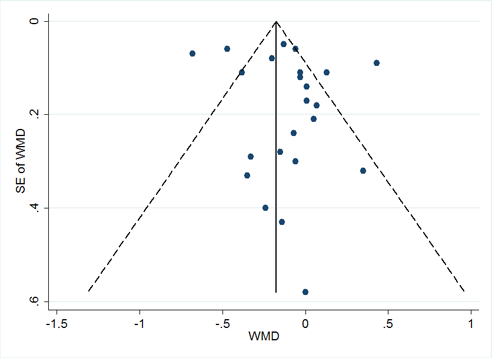

Supplement: Figure S5 — Funnel plot for publication bias (n-3 PUFAs supplementation and TNF-α in chronic non-autoimmune disease). SE, standard error; WMD, weighted mean difference. (TIF) [file pone.0088103.s005.tif]

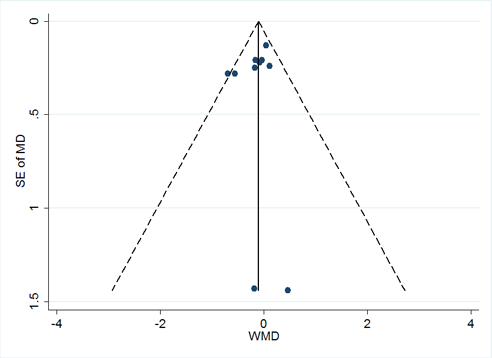

Supplement: Figure S6 — Funnel plot for publication bias (n-3 PUFAs from dietary intake and CRP in chronic non-autoimmune disease). SE, standard error; WMD, weighted mean difference. (TIF) [file pone.0088103.s006.tif]

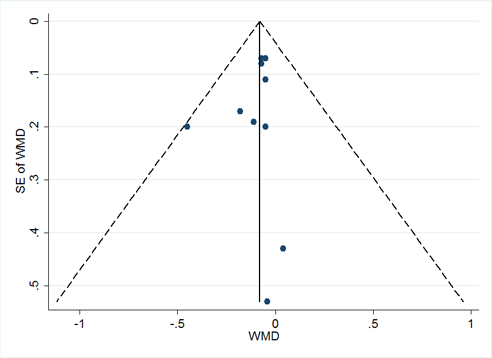

Supplement: Figure S7 — Funnel plot for publication bias (n-3 PUFAs from dietary intake and IL-6 in chronic non-autoimmune disease). SE, standard error; WMD, weighted mean difference. (TIF) [file pone.0088103.s007.tif]

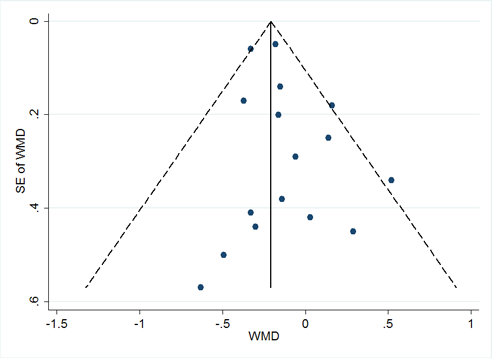

Supplement: Figure S8 — Funnel plot for publication bias (n-3 PUFAs supplementation and CRP in healthy subjects). SE, standard error; WMD, weighted mean difference. (TIF) [file pone.0088103.s008.tif]

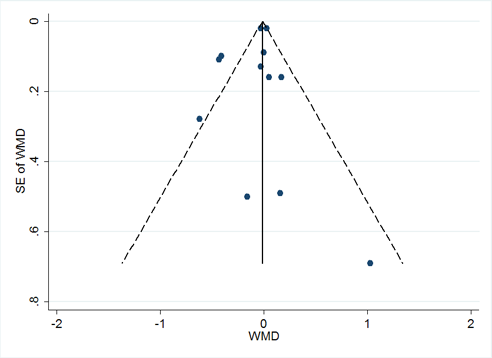

Supplement: Figure S9 — Funnel plot for publication bias (n-3 PUFAs supplementation and IL-6 in healthy subjects). SE, standard error; WMD, weighted mean difference. (TIF) [file pone.0088103.s009.tif]
