# Supplementary material for: Effect of Marine-Derived n-3 Polyunsaturated Fatty Acids on C-Reactive Protein, Interleukin 6 and Tumor Necrosis Factor α: A Meta-Analysis
Source: PLoS One. 2014 Feb 5;9(2):e88103. doi: 10.1371/journal.pone.0088103 (PMC3914936; doi:10.1371/journal.pone.0088103)
Supplement: Figure S2 — Judgements about each risk of bias item for each included study. Red ball, high risk of bias; yellow ball, unclear risk of bias; green ball, no risk of bias. (PDF) [file pone.0088103.s002.pdf]

	Random sequence generation (selection bias)	Allocation concealment (selection bias)	Binding of participants and personnel (performance bias)	Binding of outcome assessment (detection bias)	Incomplete outcome data (attrition bias)	Selective reporting (reporting bias)
Barbosa et al.2003	+	?	?	?	+	+
Bent et al.2011	+	+	+	+	+	+
Bowden et al.2009	+	?	+	?	−	+
Bragt et al.2012	+	?	+	?	−	+
Browning et al.2007	+	?	?	?	−	+
Chan et al.2002	+	?	+	?	+	+
Chiang et al.2012	+	?	−	?	?	+
Ciubotaru et al.2003	+	?	+	?	?	+
Damsgaard et al.2008	+	+	+	+	?	+
Daud et la.2012	+	?	+	?	+	+
De Mello et al.2009	+	?	−	?	−	+
Derosa et al.2009	+	+	+	+	−	+
Derosa et al.2012	+	+	+	+	−	+
Deutsch et al.2007	+	?	+	?	+	+
Engler et al.2004	+	?	+	?	+	+
Faghihi et al.2012	+	?	+	?	?	+
Faxen Irving et al.2009	+	+	+	?	−	+
Freund-Levi et al.2009	+	+	+	?	?	+
Fujioka et al.2006	+	?	+	?	?	+
Gammelmark et al.2012	+	?	+	?	?	+
Geelen et al.2004	+	?	+	?	+	+
Jones et al.2007	+	?	+	?	−	+
Kabir et al.2007	+	?	+	?	−	+
Kiecolt-Glaser et al.2011	+	+	+	+	+	+
Kiecolt-Glaser et al.2012	+	+	+	+	+	+
Koh et al.2012	+	+	−	?	+	+
Kolahi et al.2010	+	?	+	?	?	+
Kooshki et al.2011	+	?	+	?	+	+
Krebs et al.2006	+	?	+	?	+	+
Krysiak et al.2011 a	+	?	?	?	−	+
Krysiak et al.2011 b	?	?	?	?	−	+
Krysiak et al.2012 a	+	?	?	?	+	+
Krysiak et al.2012 b	?	?	?	?	+	+
Lenn et al.2002	+	?	?	?	?	+
Lindqvist et al.2007	+	?	−	+	+	+
Mackay et al.2012	+	+	+	+	?	+
Madsen et al.2003	+	?	+	?	+	+
Madsen et al.2007	+	?	+	?	+	+
Malekshahi et al.2012	+	?	+	?	+	+
Mann et al.2010	+	?	+	?	+	+
Mocking et al.2012	+	+	+	+	−	+
Moertl et al.2011	+	+	+	?	+	+
Mohammadi et al.2012	+	?	+	?	+	+
Mori et al.2003	+	?	+	?	+	+
Mori et al.2009	+	+	+	?	−	+
Munro et al.2012	+	?	+	?	−	+
Murphy et al.2007	+	?	+	?	+	+
Nodari et al.2009	+	?	+	?	+	+
Nodari et al.2011	+	?	+	?	+	+
Ottestad et al.2012	+	+	+	+	−	+
Pooya et al.2010	+	?	+	?	−	+
Pot et al.2009	+	+	+	?	+	+
Ramel et al.2010	+	?	−	?	+	+
Rizza et al.2009	+	?	+	?	+	+
Sabour et al.2012	+	?	+	?	−	+
Saifullah et al.2007	+	?	+	?	−	+
Sanders et al.2006	+	+	+	+	+	+
Shahbakhti et al.2004	+	?	+	?	+	+
Skulas-Ray et al.2011	+	?	+	?	−	+
Theobald et al.2007	+	?	+	?	+	+
Thusgaard et al.2009	+	?	+	?	−	+
Tierney et al.2011	+	?	−	?	?	+
Vega-Lopez et al.2004	+	?	+	?	+	+
Watanabe et al.2009	+	?	+	?	+	+
Wong et al.2010	+	+	+	+	+	+
Wright et al.2008	+	+	+	+	+	+
Zhang et al.2012	+	?	−	?	+	+
Zhao et al.2009	+	?	−	?	+	+
