# Supplementary material for: Effect of Marine-Derived n-3 Polyunsaturated Fatty Acids on C-Reactive Protein, Interleukin 6 and Tumor Necrosis Factor α: A Meta-Analysis
Source: PLoS One. 2014 Feb 5;9(2):e88103. doi: 10.1371/journal.pone.0088103 (PMC3914936; doi:10.1371/journal.pone.0088103)
Supplement: Table S1 — Baseline of inflammatory markers (log-transformed) and mean changes of phospholipid n-3 PUFAs in plasma/serum. (DOC) [file pone.0088103.s010.doc]

**Table S1.** Baseline of inflammatory markers (log-transformed) and mean changes of phospholipid n-3 PUFAs in plasma/serum.

| Study | Biomarkers assessed | Baseline (log-transformed) | | | Changes of PL n-3 PUFAs in plasma/serum (%) | | |
| --- | --- | --- | --- | --- | --- | --- | --- |
|  |  | CRP (mg/L) | IL-6 (pg/mL) | TNF-α (pg/mL) | EPA | DHA | Total n-3 PUFAs |
| Barbosa et al.2003 | CRP | 3.99 | UN | UN | UN | UN | UN |
| Bent et al.2011 | IL-6, TNF-α | UN | 1.28 | 1.62 | UN | UN | UN |
| Bowden et al.2009 | CRP | 4.8 | UN | UN | UN | UN | UN |
| Bragt et al.2012 | CRP, IL-6, TNF-α | UN | UN | UN | UN | UN | UN |
| Browning et al.2007 A | CRP, IL-6 | 0.75 | 0.78 | UN | UN | UN | UN |
| Browning et al.2007 B | CRP, IL-6 | 0.1 | 0.09 | UN | UN | UN | UN |
| Chan et al.2002 | CRP, IL-6, TNF-α | 0.73 | -1.16 | -0.81 | UN | UN | UN |
| Chiang et al.2012 | CRP, IL-6, TNF-α | UN | UN | UN | UN | UN | UN |
| Ciubotaru et al.2003 A | CRP, IL-6 | 0.76 | 0.1 | UN | UN | UN | UN |
| Ciubotaru et al.2003 B | CRP, IL-6 | 0.65 | 0.18 | UN | UN | UN | UN |
| Damsgaard et al.2008 A | CRP, IL-6 | -1.02 | 0 | UN | UN | UN | UN |
| Damsgaard et al.2008 B | CRP, IL-6 | -1.29 | -0.19 | UN | UN | UN | UN |
| Daud et la.2012 | CRP | 4.05 | UN | UN | UN | UN | UN |
| De Mello et al.2009 A | CRP, IL-6, TNF-α | -0.31 | 0.44 | 0.24 | 1.65 | 0.15 | 1.69 |
| De Mello et al.2009 B | CRP, IL-6, TNF-α | 0.23 | 0.34 | 0.3 | -0.51 | 0 | 0.22 |
| Derosa et al.2009 | CRP | 2.61 | UN | UN | UN | UN | UN |
| Derosa et al.2012 | CRP, IL-6, TNF-α | 0.53 | 0.43 | 6.99 | UN | UN | UN |
| Deutsch et al.2007 | CRP | 3.54 | UN | UN | UN | UN | UN |
| Engler et al.2004 | CRP | -0.76 | UN | UN | UN | UN | UN |
| Faghihi et al.2012 | CRP | 3.25 | UN | UN | UN | UN | UN |
| Faxen Irving et al.2009 | CRP, IL-6 | -0.11 | -0.32 | UN | UN | UN | UN |
| Freund-Levi et al.2009 | CRP, IL-6, TNF-α | -0.26 | -0.29 | 3.84 | UN | UN | UN |

**Table S1.** Continued

| Study | Biomarkers assessed | Baseline (log-transformed) | | | Changes of PL n-3 PUFAs in plasma/serum (%) | | |
| --- | --- | --- | --- | --- | --- | --- | --- |
|  |  | CRP (mg/L) | IL-6 (pg/mL) | TNF-α (pg/mL) | EPA | DHA | Total n-3 PUFAs |
| Fujioka et al.2006 | CRP | -1.12 | UN | UN | UN | UN | UN |
| Gammelmark et al.2012 | CRP, IL-6, TNF-α | 0.42 | 1.14 | 0.09 | UN | UN | UN |
| Geelen et al.2004 | CRP | 0.26 | UN | UN | UN | UN | UN |
| Jones et al.2007 | CRP, IL-6, TNF-α | UN | UN | UN | UN | UN | UN |
| Kabir et al.2007 | IL-6, TNF-α | UN | 0.71 | 0.8 | UN | UN | UN |
| Kiecolt-Glaser et al.2011 | IL-6, TNF-α | UN | UN | UN | UN | UN | UN |
| Kiecolt-Glaser et al.2012 A | IL-6, TNF-α | UN | 0.87 | 0.69 | UN | UN | UN |
| Kiecolt-Glaser et al.2012 B | IL-6, TNF-α | UN | 0.98 | 0.71 | UN | UN | UN |
| Koh et al.2012 | CRP | 0.14 | UN | UN | UN | UN | UN |
| Kolahi et al.2010 | CRP, TNF-α | 3.96 | UN | 2.64 | UN | UN | UN |
| Kooshki et al.2011 | CRP, IL-6, TNF-α | 0.53 | 1.85 | 2.62 | UN | UN | UN |
| Krebs et al.2006 | CRP, IL-6, TNF-α | 0.72 | 0.62 | -0.03 | UN | UN | UN |
| Krysiak et al.2011 a | CRP | 1.16 | UN | UN | UN | UN | UN |
| Krysiak et al.2011 b | CRP | 1.09 | UN | UN | UN | UN | UN |
| Krysiak et al.2012 a | CRP | 0.35 | UN | UN | UN | UN | UN |
| Krysiak et al.2012 b | CRP | 0.49 | UN | UN | UN | UN | UN |
| Lenn et al.2002 | IL-6, TNF-α | UN | 3.38 | 0.6 | UN | UN | UN |
| Lindqvist et al.2007 | CRP | 0.66 | UN | UN | UN | UN | UN |
| Mackay et al.2012 A | CRP, IL-6 | 0.81 | -0.11 | UN | 1.08 | 1.09 | 2.17 |
| Mackay et al.2012 B | CRP, IL-6 | 0.95 | -0.14 | UN | 0.88 | 1.42 | 2.3 |
| Madsen et al.2003 A | CRP | -0.38 | UN | UN | UN | UN | UN |
| Madsen et al.2003 B | CRP | -0.09 | UN | UN | UN | UN | UN |

**Table S1.** Continued

| Study | Biomarkers assessed | Baseline (log-transformed) | | | Changes of PL n-3 PUFAs in plasma/serum (%) | | |
| --- | --- | --- | --- | --- | --- | --- | --- |
|  |  | CRP (mg/L) | IL-6 (pg/mL) | TNF-α (pg/mL) | EPA | DHA | Total n-3 PUFAs |
| Madsen et al.2007 A | CRP | -0.34 | UN | UN | UN | UN | UN |
| Madsen et al.2007 B | CRP | 1.49 | UN | UN | UN | UN | UN |
| Malekshahi et al.2012 | CRP, TNF-α | 2.75 | UN | 3.62 | UN | UN | UN |
| Mann et al.2010 A | CRP | 2.23 | UN | UN | UN | UN | UN |
| Mann et al.2010 B | CRP | 3.28 | UN | UN | UN | UN | UN |
| Mocking et al.2012 | CRP, IL-6, TNF-α | 1.26 | 0.79 | 0.79 | 1.45 | -0.26 | 1.66 |
| Moertl et al.2011 A | IL-6, TNF-α | UN | 1.15 | 0.81 | UN | UN | UN |
| Moertl et al.2011 B | IL-6, TNF-α | UN | 1.34 | 0.94 | UN | UN | UN |
| Mohammadi et al.2012 | CRP | 0.75 | UN | UN | UN | UN | UN |
| Mori et al.2003 A | CRP, IL-6, TNF-α | 0.85 | 0.72 | 2.93 | 1.11 | 6.71 | 7.14 |
| Mori et al.2003 B | CRP, IL-6, TNF-α | 0.42 | 0.56 | 3.05 | 8.79 | -0.25 | 10.51 |
| Mori et al.2009 | CRP | 0.51 | UN | UN | UN | UN | UN |
| Munro et al.2012 | CRP, IL-6, TNF-α | 1.12 | 0.64 | -0.14 | UN | UN | UN |
| Murphy et al.2007 | CRP | 1.27 | UN | UN | UN | UN | UN |
| Nodari et al.2009 | IL-6, TNF-α | UN | 1.89 | 2.94 | UN | UN | UN |
| Nodari et al.2011 | IL-6, TNF-α | UN | 2.25 | 3.03 | UN | UN | UN |
| Ottestad et al.2012 | CRP | -0.33 | UN | UN | UN | UN | UN |
| Pooya et al.2010 | CRP | 1.07 | UN | UN | UN | UN | UN |
| Pot et al.2009 | IL-6, TNF-α | UN | 1.66 | 1.01 | UN | UN | UN |
| Ramel et al.2010 AF | CRP, IL-6 | 0.68 | 0.27 | UN | UN | UN | UN |
| Ramel et al.2010 AM | CRP, IL-6 | 0.27 | 0.11 | UN | UN | UN | UN |
| Ramel et al.2010 BF | CRP, IL-6 | 0.73 | 0.3 | UN | UN | UN | UN |

**Table S1.** Continued

| Study | Biomarkers assessed | Baseline (log-transformed) | | | Changes of PL n-3 PUFAs in plasma/serum (%) | | |
| --- | --- | --- | --- | --- | --- | --- | --- |
|  |  | CRP (mg/L) | IL-6 (pg/mL) | TNF-α (pg/mL) | EPA | DHA | Total n-3 PUFAs |
| Ramel et al.2010 BM | CRP, IL-6 | 0.25 | 0.14 | UN | UN | UN | UN |
| Ramel et al.2010 CF | CRP, IL-6 | 0.71 | 0.14 | UN | UN | UN | UN |
| Ramel et al.2010 CM | CRP, IL-6 | 0.31 | 0.15 | UN | UN | UN | UN |
| Rizza et al.2009 | CRP, IL-6, TNF-α | 0.18 | 0.3 | 1.98 | UN | UN | UN |
| Sabour et al.2012 | IL-6, TNF-α | UN | 4.06 | 3.52 | UN | UN | UN |
| Saifullah et al.2007 | CRP | 2.38 | UN | UN | UN | UN | UN |
| Sanders et al.2006 | CRP | 0.03 | UN | UN | UN | UN | UN |
| Shahbakhti et al.2004 | IL-6, TNF-α | UN | -0.16 | -0.07 | UN | UN | UN |
| Skulas-Ray et al.2011 | CRP, IL-6, TNF-α | 0.05 | -0.46 | 0.17 | UN | UN | UN |
| Theobald et al.2007 | CRP, IL-6 | -1.03 | 6.88 | UN | UN | UN | UN |
| Thusgaard et al.2009 | CRP | 0.77 | UN | UN | UN | UN | UN |
| Tierney et al.2011 | CRP, IL-6, TNF-α | 1.43 | 1.31 | 1.23 | UN | UN | UN |
| Vega-Lopez et al.2004 | CRP | 0.2 | UN | UN | UN | UN | UN |
| Watanabe et al.2009 | CRP | -0.95 | UN | UN | -0.8 | -0.5 | -1.3 |
| Wong et al.2010 | CRP | -0.07 | UN | UN | UN | UN | UN |
| Wright et al.2008 | CRP | 1.29 | UN | UN | UN | UN | UN |
| Zhang et al.2012 A | CRP, IL-6, TNF-α | 0.5 | 5.54 | 2.55 | 0.8 | 1.3 | 2.2 |
| Zhang et al.2012 B | CRP, IL-6, TNF-α | 0.62 | 5.51 | 2.57 | 1 | 1.1 | 2.3 |
| Zhang et al.2012 C | CRP, IL-6, TNF-α | 0.54 | 5.46 | 2.53 | 0.7 | 1 | 2.1 |
| Zhao et al.2009 | CRP, IL-6, TNF-α | 2.8 | 2.18 | 4.07 | UN | UN | UN |

T, intervention in treatment group; C, intervention in control group. Studies marked with different capital letters were independent comparisons from the same study; studies marked with different minuscules were different studies with the same first author and published in the same year. UN, unclear; PL, phospholipid.
