# Supplementary material for: Effect of Marine-Derived n-3 Polyunsaturated Fatty Acids on C-Reactive Protein, Interleukin 6 and Tumor Necrosis Factor α: A Meta-Analysis
Source: PLoS One. 2014 Feb 5;9(2):e88103. doi: 10.1371/journal.pone.0088103 (PMC3914936; doi:10.1371/journal.pone.0088103)
Supplement: Text S1 — Protocol for meta-analysis to assess the effect of marine-derived n-3 polyunsaturated fatty acids on CRP, IL-6 and TNF-α. (DOC) [file pone.0088103.s012.doc]

**Protocol for meta-analysis to assess the effect of marine-derived n-3 polyunsaturated fatty acids on CRP, IL-6 and TNF-α**

**Objective**

To systematically evaluate the effect of marine-derived n-3 PUFAs from supplementation or dietary intake on fasting blood levels of CRP, IL-6 and TNF-α in subjects with chronic disease and healthy subjects.

**Inclusion Criteria**

1. Having a randomized placebo controlled design;

2. Reported data on fasting blood levels of TNF-α, IL-6, or CRP;

3. Having a drop-out rate less than 30%;

4. Recruited subjects with chronic disease (chronic autoimmune or non-autoimmune disease) or healthy subjects.

**Searching Methods**

The following database will be searched: Web of Science, PubMed, Embase and Medline. Hand-searching of the bibliographic sections of all relevant articles and recent reviews was also undertaken. Following Medical Subject Headings terms or key words were searched:

#1: “n-3 polyunsaturated fatty acid” OR “omega-3 polyunsaturated fatty acid” OR “ω-3 fatty acid” OR “omega-3 fatty acid” OR “polyunsaturated fatty acid” OR “eicosapentaenoic acid” OR “docosahexaenoic acid” OR “fish oil”

#2: “interleukin 6” OR “interleukin” OR “tumor necrosis factor” OR “tumor necrosis factor alpha” OR “TNF-α” OR “cytokine” OR “C-reactive protein” OR “CRP” OR “inflammation”

#1 AND #2

The search was restricted to studies published in any language from 1950 to 2013.

**Data Extraction and Quality Assessment**

Study selection and data extraction will be undertaken independently by two investigators, with discrepancies resolved by consensus. The data collected includes the first author’s name, year of publication, sample size, age, BMI sex ratio (male/total subjects) and healthy status of subjects, intervention methods, study design (parallel-group, crossover or factorial), daily dose of n-3 PUFAs, duration of follow-up, and data of fasting blood level of TNF-α, IL-6 and CRP.

Cochrane criteria will be used to assess the quality of studies included in the present meta-analysis, including sequence generation, allocation concealment, blinding of participants, personnel and outcome assessors, incomplete outcome data and selective outcome reporting .

**Meta-analysis**

Studies recruiting subjects with chronic autoimmune disease, studies recruiting subjects with chronic non-autoimmune disease and studies recruiting healthy subjects will be included into different meta-analyses. In addition, We will also separate studies using n-3 polyunsaturated fatty acids from supplementation (seafood oils or highly purified n-3 polyunsaturated fatty acids) as active treatment with studies using n-3 polyunsaturated fatty acids from dietary intake (fish intake), and included them into different meta-analyses.

Weighted mean difference will be used as the effect size. A random-effect model will be used to pool study-specific effect sizes, and a P value < 0.05 is considered to be statistically significant. Heterogeneity will be assessed using the chi-square method. I^2^ > 50% indicated significant heterogeneity . Subgroup analysis will be undertaken to explore the sources of heterogeneity and their influence on effect size according to the difference in study design, placebo, duration, daily dose of n-3 PUFAs, and age, sex ratio (male/total subjects) and body mass index (BMI) of subjects; the cut-off points for duration, daily dose and age are their medians; 0.5 is used as the cut-off point for sex ratio; 30 kg/m^2^ is used as the cut-off points for BMI. For meta-analysis in subjects with chronic diseases, subgroup analysis will also be conducted according to difference in types of diseases. Meta-regression will be conducted to explore whether subgroup difference is significant, and whether there is significant linear relationship (P for coefficient < 0.05) between effect size and continuous confounding variables (duration, daily dose, age, sex ratio and BMI); the adjusted R^2^ indicated the percentage of between-study heterogeneity that can be explained by the confounding variables. If two or more confounding factors show significant influence on the effect size (P for subgroup difference or coefficient < 0.05), a joint test will be conducted to explain heterogeneity caused by these confounding factors by including them into a single meta-regression model simultaneously ; the adjusted R^2^ of the overall model indicates the percentage of between-study heterogeneity that can be explained by these confounding factors . If no significant linear relationship is observed between effect size and continuous confounding variables, restricted cubic spline analysis will be used to test potential cubic relationship between them (3 knots) . We will also conduct sensitivity analysis to assessing the effect of bias on the pooled effect size by excluding studies having a high risk of bias for two or more validity criteria and then reanalyzing the remaining data. Publication bias will be evaluated by funnel plot method. If significant publication bias is observed, trim-and-fill method will be used to adjust the pooled effect size.

**Reference**

1. Higgins JPT, Green S (2011) Cochrane Handbook for Systematic Reviews of Interventions Version 5.1.0 [updated March 2011]. Available: http://www.cochrane-handbook.org. Accessed 5 May 2012.

2. Hooper L, Summerbell CD, Thompson R, Sills D, Roberts FG, et al. (2012) Reduced or modified dietary fat for preventing cardiovascular disease. Cochrane Database Syst Rev 5: CD002137.

3. Harbord RM, Higgins JP (2008) Meta-regression in Stata. Meta 8: 493-519.

4. Zheng J-S, Hu X-J, Zhao Y-M, Yang J, Li D (2013) Intake of fish and marine n-3 polyunsaturated fatty acids and risk of breast cancer: meta-analysis of data from 21 independent prospective cohort studies. BMJ 346: f3706.
